# Non-obese NAFLD had no better cardio-metabolic risk profile than obese NAFLD in type 2 diabetic patients

**DOI:** 10.1186/s12933-022-01648-9

**Published:** 2022-10-14

**Authors:** Ziyin Zhang, Lu Zhang, Wangyan Jiang, Tingting Du, Gang Yuan

**Affiliations:** 1 Department of Endocrinology, Tongji Hospital, Tongji Medical College, Huazhong University of Science and Technology, 430030 Wuhan, China; 2 Branch of National Clinical Research Center for Metabolic Diseases, Wuhan, China

**Keywords:** Non-obese non-alcoholic fatty liver disease, Cardio-metabolic risk, Type 2 diabetes mellitus

## Abstract

**Background:**

Non-obese non-alcoholic fatty liver disease (NAFLD) has been reported to share clinical outcomes with its obese counterpart in the general population. However, conflicting results have been observed regarding the cardio-metabolic risk profile of non-obese NAFLD as compared to obese NAFLD. Moreover, in the context of type 2 diabetes mellitus (T2DM), this issue has been even less addressed. We hence aimed to examine the association of NAFLD with the cardio-metabolic risk profile in patients with T2DM according to their obesity status.

**Methods:**

A total of 2,708 patients with T2DM who were hospitalized between June 2018 and May 2021 were cross-sectionally assessed.

**Results:**

The prevalence of NAFLD was 49.3%. NAFLD was found in 34.1% of non-obese patients and 66.0% of obese patients. Non-obese NAFLD patients had more and worse metabolic disorders than obese patients without NAFLD in both men and women. Comparable cardio-metabolic risk profiles were noted between non-obese and obese NAFLD subjects. The associations of worse cardio-metabolic risk profiles with NAFLD were overall stronger in non-obese than in obese subjects among women with T2DM, while more pronounced in obese than in non-obese subjects among men with T2DM.

**Conclusion:**

In patients with T2DM, non-obese NAFLD had no better cardio-metabolic risk profile than obese NAFLD. The associations of metabolic disorders with NAFLD were stronger in non-obese than in obese patients in women patients with T2DM.

**Supplementary Information:**

The online version contains supplementary material available at 10.1186/s12933-022-01648-9.

## Background

Non-alcoholic fatty liver disease (NAFLD) is a leading cause of chronic liver disease, affecting around 25.2% of the global population [1]. It is highly prevalent in type 2 diabetic patients, with a global prevalence of 55.5% among them [2]. Type 2 diabetic patients with NAFLD have worse glycemic control and develop diabetic-related complications more rapidly than those without NAFLD [3, 4]. Vice versa, the presence of type 2 diabetes mellitus (T2DM) increases the burden of NAFLD due to an increased risk of progression to steatohepatitis, fibrosis, cirrhosis, and hepatocellular carcinoma [3].

Although NAFLD patients are classically seen with overweight or obesity, this entity can also affect non-obese individuals: it was reported that the global prevalence of non-obese NAFLD was over 40% among the NAFLD population and nearly 20% in non-obese population [5]. Reports showed that non-obese NAFLD was more prevalent in diabetic patients compared with the general population [5], suggesting that non-obese NAFLD contributes to a large share of disease burden of diabetes. Emerging evidence showed that non-obese NAFLD patients have comparable or even worse clinical outcomes than obese-NAFLD patients in the general population [5, 6]. However, studies on the cardio-metabolic risk profiles of non-obese NAFLD patients when compared with obese NAFLD have shown inconsistent results [6–9], and this topic has been even less discussed in the T2DM population. Therefore, we aimed to investigate the cardio-metabolic risk profiles in patients with T2DM according to their obesity and NAFLD status.

## Methods

### Study design and population

This cross-sectional study included 3,011 T2DM patients hospitalized in the Department of Endocrinology, Tongji Hospital, Tongji medical college, Huazhong University of Science and Technology (Wuhan, China) between 2018 and 2021. T2DM was diagnosed according to the 2022 American Diabetes Association criteria [10]. We excluded 154 patients with a positive hepatitis B surface antigen or hepatitis C antibody, 2 with excessive alcohol intake (> 30 g/day for men and > 20 g/day for women), and 149 with missing data on liver ultrasonography. Patients with hereditary causes of liver disease such as Wilson disease, and hereditary hemochromatosis, or taking drugs such as amiodarone, and corticosteroid that may incur fatty liver were also excluded. The remaining available 2,708 patients were included in the present analyses. According to the Private Information Protection Law, information that might identify subjects was safeguarded by the Computer Center. This study was approved by the institutional review board of Tongji Hospital. Because we only retrospectively accessed a de-identified database for purposes of analysis, informed consent requirement was exempted by the institutional review board.

### Clinical measurements

Patients’ data including age, sex, height, weight, histories of current and previous illness, and medical treatments were obtained from medical records. Height, weight, waist circumference (WC), and blood pressure (BP) were measured according to standardized protocols from the World Health Organization (WHO). Patients’ seated BP was measured twice for every 5 min on the right arm after 5 min of rest with a sphygmomanometer. The mean of the two readings was used in data analysis. Body mass index (BMI) was calculated as weight (in kilograms) divided by the square of height (in meters).

Overnight fasting (for at least 8 h) blood samples were collected from the antecubital vein of each patient. The first urine specimens in the morning were collected. All blood and urine specimens were tested immediately after collection. Glycated hemoglobin (HbA1c) was measured using high performance liquid chromatography (D-10™; Bio‐Rad Laboratories, Hercules, CA, USA). Fasting plasma glucose (FPG), triglycerides (TG), total cholesterol (TC), high‐density lipoprotein cholesterol (HDL-C), low‐density lipoprotein cholesterol (LDL-C), alanine aminotransferase (ALT), aspartate aminotransferase (AST), uric acid, and creatinine were measured on an autoanalyzer (Cobas C8000, Roche, Mannheim, Germany). Insulin levels were measured by chemiluminescent immunometric assay (Cobas e601; Roche Diagnostics Ltd., Indianapolis, IN, USA). Urinary albumin was measured using the immunoturbidimetric method (Cobas C8000; Roche, Mannheim, Germany). Hepatitis viral antigens/antibodies were detected with corresponding Architect reagents (Architect i2000, Abbott Diagnostics, Abbott Park, IL). Homeostatic Model Assessment for Insulin Resistance (HOMA-IR) was calculated as FPG (mmol/L) × fasting insulin (FINS) (µU/mL)/22.5. Non-HDL-C was calculated as TC - HDL-C. Estimated glomerular filtration rate (eGFR) was calculated using the formula: eGFR = 186.3 × sCr^− 1.154^ × age^− 0.203^ (× 0.742 in women).

The area of subcutaneous adipose tissue (SAT) and visceral adipose tissue (VAT) was measured by bio-electrical impedance analyses.

### Ultrasonography

Ultrasound tests were performed by certified sonographers using a high-resolution, real-time scanner (model SSD-2000; Aloka Co., Ltd., Tokyo Japan). Certified radiologists used standard criteria in evaluating the presence or absence of hepatic fat [11]. Generally, liver steatosis was defined as the presence of stronger echoes in the hepatic parenchyma compared with echoes in the kidney or spleen parenchyma [11]. Severity of steatosis was defined as mild, moderate, and severe.

Left ventricular (LV) mass was measured by 2-dimentional echocardiography. Left ventricular end diastolic and end systolic volumes and ejection fraction (EF) were measured at the apical two chamber and four-chamber views when patients were at rest. Transmitral peak early diastolic velocity (E) and peak late diastolic velocity (A) were measured by pulsed-wave Doppler echocardiography. Early annular diastolic tissue velocity (e’) was measured by pulsed-wave tissue Doppler echocardiography.

Carotid intima-media thickness (IMT) was measured on the far wall of the common carotid artery using a LOGIC E9 ultrasound scanner (GE Healthcare, Milwaukee, WI, USA) [12]. The mean of left and right IMT was used in the analyses.

### Definitions

According to World Health Organization Asia-Pacific guidelines [13], obesity was defined as BMI ≥ 25 kg /m^2^. In the sensitivity analysis, a BMI value of 23 kg/m^2^ was used to define normal weight according to the same guidelines [13].

According to the American Diabetes Association (ADA) criteria, poor glycemic control was defined as HbA1c level ≥ 7.0%; poor BP control as BP ≥ 130/80 mmHg; poor cholesterol control as LDL-C level ≥ 100 mg/dL; poor TG control as TG level ≥ 150 mg/dL; poor HDL-C control as HDL-C level ≤ 40/50 mg/dL for men/women [14, 15].

Heart failure with preserved ejection fraction (HFpEF) was defined as EF ≥ 50% with either (1) E/A < 0.8, E/e’ < 8, and peak e’ < 10 cm/s, or (2) 0.8 < E/A < 1.5, 8 < E/e’ < 14, and peak e’ < 8 cm/s, or (3) E/A > 1.5, E/e’ >14 and peak e’ < 5 cm/s [16].

Left ventricular hypertrophy (LVH) was defined as LV mass/body surface area (BSA) > 115 g/m^2^ in men and > 95 g/m^2^ in women [17]. The formulae used to calculate LV mass and BSA have been previously described [17, 18].

Fibrosis was defined by the Fibrosis-4 (FIB-4) index or NAFLD Fibrosis Score (NFS) as previously described [19].

### Statistical analyses

All statistical analyses were conducted using R Language, version 4.1.1 (The R Foundation for Statistical Computing, Vienna, Austria). Continuous variables were presented as means (SDs) or medians (IQRs) depending on their distribution. Categorical variables were presented as numbers (percentages). Differences in continuous variables between groups were tested with ANOVA or Kruskal-Wallis test. Differences in categorical variables were tested with χ^2^ test. Tukey or Benjamini-Hochberg corrections were made based on multiple different comparisons. Age-, WC-, smoking status-, T2DM duration-, anti-diabetic drugs-, anti-hypertensive drugs-, and lipid-lowering drugs-adjusted means of cardio-metabolic risk factors were calculated using generalized linear models. Crude and adjusted odds ratios (ORs) for cardio-metabolic risk profiles were calculated using logistic regression analyses, with non-obese non-NAFLD (lean non-NAFLD in sensitivity analyses) as the reference. Potentially confounding variables such as age, WC, smoking status, HbA1c, BP, LDL-C, duration of T2DM, anti-diabetic drugs, anti-hypertensive drugs, and lipid-lowering drugs were adjusted. P values < 0.05 were considered statistically significant.

## Results

Of the 2,708 diabetic patients included, the mean age was 52.8 (12.9) years. 1,705 (63.0%) were men, 1,335 (49.3%) were with NAFLD and 1,289 (47.6%) were obese. NAFLD was found in 484 (34.1%) of non-obese patients and 851 (66.0%) of obese patients.

Characteristics of the study population according to their obesity and NAFLD status were shown in Table 1. Information on anti-diabetic drug use was shown in Supplementary Table 1. 193 (7.13%), 39 (1.44%), and 115 (4.25%) patients used thiazolidinediones (TZDs), sodium-glucose cotransporter-2 inhibitors (SGLT-2Is), and glucagon-like peptide-1 receptor agonists (GLP-1RAs), respectively. Compared to non-obese patients without NAFLD, non-obese NAFLD patients were younger and had higher diastolic BP, HbA1c, HOMA-IR, TC, TG, non-HDL-C, SAT, VAT, visceral to subcutaneous adipose tissue ratio (VSR), and lower HDL-C levels, but not statistically different WC, systolic BP, FPG, LDL-C, and IMT levels; despite no difference in the prevalence of LVH, non-obese NAFLD patients were more likely to have poor BP, HbA1c, TG, LDL-C, and HDL-C control and suffer from HFpEF. Compared to obese patients without NAFLD, non-obese NAFLD patients were younger, and had lower WC, systolic BP, SAT, and VAT levels, but higher HbA1c, TC, TG, LDL-C, and non-HDL-C levels; diastolic BP, FPG, HOMA-IR HDL-C, IMT, and VSR levels were comparable between these two groups; non-obese NAFLD patients were more likely to have poor HbA1c, TG, and LDL-C control, but less likely to suffer from LVH; the prevalence of poor BP and HDL-C control and the prevalence of HFpEF were comparable between these two groups. Compared to obese NAFLD patients, non-obese NAFLD patients were older, and had lower WC, systolic BP, diastolic BP, HOMA-IR, TG, SAT, VAT, and higher HDL-C levels; HbA1c, TC, LDL-C, IMT, and VSR levels were comparable between these two groups; the prevalence of poor HbA1c and LDL-C control, HFpEF, and history of cardiovascular diseases (CVD, including coronary heart disease and stroke) were similar between non-obese NAFLD and obese NAFLD patients. Obese NAFLD patients were more likely to have moderate and severe liver steatosis than their non-obese counterparts.

**Table 1:** Characteristics of the study population (n = 2708) according to obesity and NAFLD status

	Without NAFLD		NAFLD	
	Non-obese (n = 935)	Obese (n = 438)	Obese (n = 851)	Non-obese (n = 484)
Men (%)	524 (56.0)	292 (66.7) ^*^	606 (71.0) ^*^	283 (58.4) ^†§^
Age (year)	55.2 (12.1)	55.7 (12.2)	48.9 (13.7) ^*†^	52.5 (12.0) ^*†§^
BMI (kg/m^2^)	22.0 (2.05)	27.4 (2.39) ^*^	28.3 (3.02) ^*†^	23.1 (1.60) ^*†§^
Waist circumstance (cm)	86.8 (26.4)	97.0 (8.43) ^*^	99.9 (9.37) ^*†^	89.3 (6.31) ^†§^
Systolic BP (mmHg)	129 (21.4)	135 (20.0) ^*^	135 (41.8) ^*^	129 (18.9) ^†§^
Diastolic BP (mmHg)	79.4 (12.2)	82.1 (11.8) ^*^	85.8 (12.3) ^*†^	82.0 (11.6) ^*§^
FPG (mmol/L)	8.37 (3.21)	8.39 (2.90)	9.19 (3.42) ^*†^	8.95 (3.50)
HOMA-IR	1.68 (0.81–3.17)	2.78 (1.35–5.12) ^*^	3.38 (1.79–5.48) ^*^	2.27 (1.25–4.09) ^*§^
HbA1c (%)	9.17 (2.54)	8.78 (2.30) ^*^	9.51 (2.24) ^*†^	9.79 (2.37) ^*†^
TC (mg/dL)	170 (45.8)	165 (44.0)	183 (51.8) ^*†^	185 (48.7) ^*†^
TG (mg/dL)	139 (92.1–213)	168 (112–272) ^*^	241 (159–390) ^*†^	218 (142–381) ^*†§^
LDL-C (mg/dL)	105 (36.9)	100 (36.2)	107 (36.9) ^†^	110 (37.8) ^†^
HDL-C (mg/dL)	43.8 (12.6)	40.0 (9.94) ^*^	36.4 (8.09) ^*†^	38.6 (9.52) ^*§^
Non-HDL-C (mg/dL)	127 (43.6)	124 (42.9)	147 (51.8) ^*†^	145 (46.5) ^*†^
ALT (U/L)	15.0 (12.0–22.0)	19.0 (13.8–27.0) ^*^	27.0 (18.0–41.0) ^*†^	21.0 (15.0–30.0) ^*†§^
AST (U/L)	17.0 (14.0–21.0)	18.0 (15.0–24.0) ^*^	21.0 (17.0–29.0) ^*†^	18.0 (15.0–24.0) ^*§^
γ-GT (U/L)	20.0 (15.0–31.0)	26.0 (19.0–38.0) ^*^	38.0 (26.0–57.0) ^*†^	31.0 (20.0–46.0) ^*†§^
Cr (µmol/L)	68.0 (56.0–85.0)	76.0 (60.5–95.0) ^*^	71.0 (60.0–83.0) ^†^	64.0 (54.0–78.2) ^*†§^
UA (µmol/L)	305 (187)	339 (108) ^*^	367 (103) ^*†^	320 (95.3) ^§^
eGFR (mL/min/1.73 m^2^)	90.8 (26.3)	85.6 (26.3) ^*^	98.2 (22.3) ^*†^	98.5 (21.9) ^*†^
UACR (mg/g)	14.1 (6.80–50.2)	17.4 (7.10–101) ^*^	15.6 (6.70–48.9) ^†^	11.9 (6.60–27.1) ^*†§^
IMT (mm)	0.77 (0.39)	0.79 (0.25)	0.75 (0.19)	0.74 (0.15)
SAT (cm^2^)	133 (43.1)	194 (43.8) ^*^	233 (73.2) ^*†^	162 (42.8) ^*†§^
VAT (cm^2^)	55.2 (32.9)	104 (41.9) ^*^	123 (45.4) ^*†^	81.5 (30.1) ^*†§^
VSR	0.36 (0.22)	0.47 (0.17) ^*^	0.49 (0.13) ^*^	0.45 (0.17) ^*^
T2DM duration (year)	7.84 (7.11)	8.44 (7.61)	4.90 (5.98) ^*†^	5.54 (6.56) ^*†^
Smoking (%)	241 (25.7)	116 (26.5)	283 (33.2) ^*†^	134 (27.7)
BP ≥ 130/80 mmHg (%)	570 (61.0)	308 (70.3) ^*^	664 (78.0) ^*†^	330 (68.2) ^*§^
HbA1c ≥ 7.0% (%)	718 (78.1)	332 (76.9)	744 (88.2) ^*†^	424 (88.5) ^*†^
TG ≥ 150 mg/dL (%)	419 (45.3)	246 (57.1) ^*^	664 (78.2) ^*†^	348 (72.5) ^*†§^
LDL-C ≥ 100 mg/dL (%)	485 (52.5)	211 (49.0)	482 (57.0) ^†^	286 (60.0) ^*†^
HDL-C ≤ 40/50 mg/dL for men/women (%)	531 (57.5)	287 (66.4) ^*^	681 (80.7) ^*†^	347 (72.6) ^*§^
HFpEF (%)	145 (16.7)	76 (18.9)	160 (20.1)	107 (23.5) ^*^
LVH (%)	131 (14.6)	78 (18.6)	105 (12.8) ^†^	51 (10.9) ^†^
Anti-diabetic drugs (%)	678 (72.5)	290 (66.2) ^*^	513 (60.3) ^*^	286 (59.1) ^*†^
SUs	186 (19.9)	76 (17.4)	132 (15.5)	81 (16.7)
Non-SUs	33 (3.53)	8 (1.83)	17 (2.00)	11 (2.27)
Biguanides	297 (31.8)	155 (35.4)	283 (33.3)	160 (33.1)
AGIs	269 (28.8)	123 (28.1)	174 (20.4) ^*†^	93 (19.2) ^*†^
TZDs	60 (6.42)	48 (11.0) ^*^	61 (7.17)	24 (4.96) ^†^
DPP-4Is	83 (8.88)	45 (10.3)	52 (6.11)	55 (11.4) ^§^
SGLT-2Is	39 (4.17)	22 (5.02)	40 (4.70)	14 (2.89)
Insulin	337 (36.0)	185 (42.2)	210 (24.7) ^*†^	117 (24.2) ^*†^
GLP-1RAs	5 (0.53)	11 (2.51) ^*^	19 (2.23) ^*^	4 (0.83)
Anti-hypertensive drugs (%)	283 (30.3)	221 (50.5) ^*^	340 (40.0) ^*†^	143 (29.5) ^†§^
Lipid-lowering drugs (%)	101 (10.8)	73 (16.7) ^*^	107 (12.6)	56 (11.6)
Degree of steatosis (%) ^a^				
Mild	-	-	267 (61.2)	205 (80.7)
Moderate	-	-	128 (29.4)	42 (16.5)
Severe	-	-	41 (9.40)	7 (2.76)
History of CVD (%)	139 (14.9)	95 (21.7) ^*^	109 (12.8) ^†^	44 (9.09) ^*†^

BMI, body mass index; BP, blood pressure; FPG, fasting plasma glucose; HOMA-IR, homeostasis model assessment of insulin resistance; HbA1c, glycated hemoglobin; TC, total cholesterol; TG, triglycerides; LDL-C, low density lipoprotein cholesterol; HDL-C, high density lipoprotein cholesterol; ALT, alanine aminotransferase; AST, aspartate aminotransferase; γ-GT, γ-glutamyl transferase; Cr, creatinine; UA, uric acid; eGFR, estimated glomerular filtration rate; UACR, urinary albumin to creatinine ratio; IMT, intima-media thickness; SAT, subcutaneous adipose tissue; VAT, visceral adipose tissue; VSR, visceral to subcutaneous adipose tissue ratio; T2DM, type 2 diabetes mellitus; HFpEF, heart failure with preserved ejection fraction; LVH, left ventricular hypertrophy; SUs, sulfonylureas; AGIs, α-glucosidase inhibitors; TZDs, thiazolidinediones; DPP-4Is, dipeptidyl peptidase-4 inhibitors; SGLT-2Is, sodium-glucose cotransporter-2 inhibitors; GLP-1RAs, glucagon-like peptide-1 receptor agonists; CVD, cardiovascular disease

Data are presented as means (SDs), medians (IQRs), or numbers (percentages) depending on their distribution

^a^ n = 690;

^*^ p < 0.05 compared with the group of non-obese without NAFLD;

^†^ p < 0.05 compared with the group of obesity without NAFLD;

^§^ p < 0.05 compared with the group of obesity with NAFLD.

**Fig. 1:**
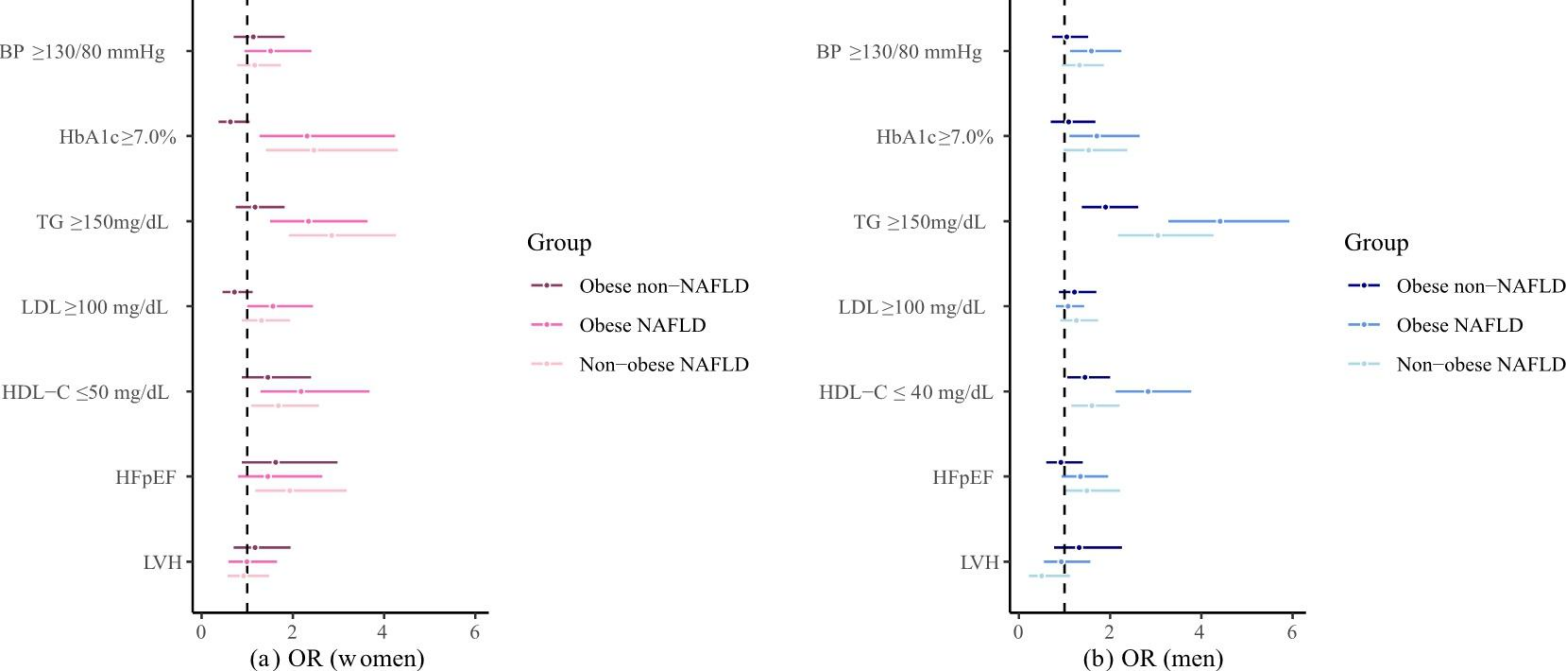
Associations of NAFLD with cardio-metabolic risk profiles according to obesity status in women and men ORs in (a) women and (b) men. BP, blood pressure; HbA1c, glycated hemoglobin; TG, triglycerides; LDL-C, low density lipoprotein cholesterol; HDL-C, high density lipoprotein cholesterol; HFpEF, heart failure with preserved ejection fraction; LVH, left ventricular hypertrophy. Data are presented as odds ratios (95% confidence intervals). Models were adjusted for age, waist circumference, smoking status, HbA1c, BP, LDL-C, duration of T2DM, anti-hypertensive drugs, and lipid-lowering drugs

Since accumulating evidence has shown sex disparities in the epidemiology, progression, and outcomes of NAFLD and T2DM [20, 21], we stratified the data by sex. In women, compared to non-obese patients without NAFLD, non-obese NAFLD patients had higher age-, WC-, and smoking status-adjusted means of HbA1c, TC, TG, non-HDL-C, and VAT and lower means of HDL-C. Compared to obese patients without NAFLD, non-obese NAFLD patients had higher adjusted means of HbA1c, TC, TG, LDL-C, and non-HDL-C; no significant difference was seen for the adjusted means of systolic and diastolic BP, FPG, HbA1c, TC, TG, LDL-C, HDL-C, non-HDL-C, and VSR between non-obese and obese NAFLD patients. However, non-obese NAFLD patients had lower SAT and VAT (Table 2). Compared to non-obese patients without NAFLD, NAFLD patients, regardless of obesity status, were more likely to suffer from poor HbA1c, TG, and HDL-C control. In the adjusted model, the association of poor TG control and HFpEF with NAFLD was stronger in non-obese patients than in obese patients (Fig. 1).

**Table 2:** Adjusted means of cardio-metabolic risk factors according to obesity and NAFLD status in women and men^a^

	Without NAFLD		NAFLD	
	Non-obese	Obese	Obese	Non-obese
**Women**	n = 412	n = 146	n = 246	n = 202
Systolic BP (mmHg)	132 (127, 137)	135 (130, 140)	135 (130, 140)	133 (128, 138)
Diastolic BP (mmHg)	77.5 (74.7, 80.4)	78.4 (75.2, 81.6)	80.8 (77.8, 83.9) ^*^	78.9 (76.1, 81.8)
FPG (mmol/L)	8.81 (7.85, 9.78)	8.15 (7.06, 9.24)	9.21 (8.18, 10.25)	9.30 (8.36, 10.23)
HbA1c (%)	8.93 (8.37, 9.49)	8.47 (7.84, 9.10)	9.30 (8.71, 9.90) ^†^	9.66 (9.10, 10.23) ^*†^
TC (mg/dL)	161 (150, 173)	153 (140, 165)	168 (156, 180) ^†^	174 (163, 186) ^*†^
TG (mg/dL)	222 (178, 265)	197 (148, 246)	260 (213, 306) ^†^	307 (263, 351) ^*†^
LDL-C (mg/dL)	94.9 (85.9, 104)	87.4 (77.3, 97.5)	98.9 (89.3, 108) ^†^	98.5 (89.4, 108) ^†^
HDL-C (mg/dL)	45.0 (42.3, 47.8)	42.8 (39.7, 45.9)	41.4 (38.5, 44.4) ^*^	40.9 (38.1, 43.7) ^*^
Non-HDL-C (mg/dL)	117 (106, 128)	109 (96.7, 121)	127 (115, 138) ^†^	133 (122, 144) ^*†^
SAT (cm^2^)	173 (113, 233)	246 (178, 315) ^*^	255 (194, 317) ^*^	203 (139, 268) ^§^
VAT (cm^2^)	58.7 (31.9, 85.4)	97.6 (67.5, 128) ^*^	112 (83.8, 140) ^*^	81.7 (53.4, 110) ^*§^
VSR	0.31 (0.13, 0.48)	0.27 (0.07, 0.47)	0.40 (0.22, 0.58) ^*^	0.36 (0.17, 0.58)
**Men**	n = 523	n = 292	n = 604	n = 282
Systolic BP (mmHg)	131 (129, 133)	131 (129, 134)	131 (129, 133)	130 (127, 132)
Diastolic BP (mmHg)	81.6 (80.1, 83.0)	82.6 (81.0, 84,1)	83.9 (82.6, 85.2)	83.5 (81.8, 85.1)
FPG (mmol/L)	8.33 (7.76, 8.90)	8.73 (8.07, 9.40)	9.38 (8.86, 9.91) ^*^	8.68 (8.03, 9.34)
HbA1c (%)	9.47 (9.20, 9.75)	9.14 (8.84, 9.44)	9.35 (9.10, 9.59)	9.55 (9.24, 9.87)
TC (mg/dL)	160 (154, 165)	163 (157, 169)	175 (170, 180) ^*†^	173 (167, 180) ^*^
TG (mg/dL)	219 (187, 250)	256 (222, 291)	354 (325, 382) ^*†^	335 (299, 372) ^*†^
LDL-C (mg/dL)	96.8 (92.4, 101)	97.0 (93.2, 102)	97.0 (93.2, 101)	101 (96.1, 106)
HDL-C (mg/dL)	38.9 (37.8, 40.0)	37.8 (36.6, 39.0)	35.6 (34.6, 36.6) ^*†^	35.4 (34.1, 36.7) ^*†^
Non-HDL-C (mg/dL)	120 (115, 125)	125 (119, 130)	139 (135, 144) ^*†^	137 (131, 143) ^*†^
SAT (cm^2^)	133 (111, 155)	189 (167, 211) ^*^	238 (219, 257) ^*†^	155 (129, 181) ^§^
VAT (cm^2^)	51.1 (39.1, 63.1)	97.5 (85.4, 110) ^*^	122 (112, 132) ^*†^	78.9 (65.7, 92.1) ^*§^
VSR	0.30 (0.24, 0.37)	0.46 (0.39, 0.52) ^*^	0.45 (0.40, 0.51) ^*^	0.42 (0.34, 0.49) ^*^

BP, blood pressure; FPG: fasting plasma glucose; HbA1c, glycated hemoglobin; TC, total cholesterol; TG, triglycerides; LDL-C, low density lipoprotein cholesterol; HDL-C, high density lipoprotein cholesterol; SAT, subcutaneous adipose tissue; VAT, visceral adipose tissue; VSR, visceral to subcutaneous adipose tissue ratio

Data are presented as means (95% confidence intervals)

^a^ Data were adjusted for age, waist circumference (except for SAT, VAT, and VSR), smoking status, T2DM duration, anti-diabetic drugs, anti-hypertensive drugs, and lipid-lowering drugs

^*^ p < 0.05 compared with the group of non-obese without NAFLD;

^†^ p < 0.05 compared with the group of obesity without NAFLD;

^§^ p < 0.05 compared with the group of obesity with NAFLD.

In men, compared to non-obese patients without NAFLD, non-obese NAFLD patients had higher adjusted means of diastolic BP, TC, TG, non-HDL-C, VAT, and VSR, and lower means of HDL-C. Compared to obese patients without NAFLD, non-obese NAFLD patients had higher HbA1c, TC, TG, and non-HDL-C levels. Non-obese NAFLD patients had comparable levels of diastolic BP, FPG, HbA1c, TC, TG, LDL-C, HDL-C, non-HDL-C, and VSR to obese NAFLD patients, however, lower SAT and VAT than obese NAFLD patients (Table 2). Compared to non-obese patients without NAFLD, NAFLD patients, regardless of obesity status, had higher risks of suffering from poor TG and HDL-C control. These associations were stronger in obese than in non-obese patients (Fig. 1). However, non-obese NAFLD patients were still 1.49 times as likely to suffer from HFpEF.

We further investigated the cardio-metabolic profile according to obesity and fibrosis status in T2DM patients with NAFLD. When applying FIB-4 index with the lower cutoff to define fibrosis, obese and non-obese NAFLD patients were comparably likely to have fibrosis (21.8% vs. 24.2%, p = 0.350). Non-obese women with fibrosis had the highest systolic BP, diastolic BP, FPG, HbA1c, and TG levels and the lowest HDL-C levels, but none of the differences were significant. Further, they were most likely to have poor BP, HbA1c, and HDL-C control. When applying NFS with the lower cutoff to define fibrosis, obese NAFLD patients were more likely to have fibrosis than non-obese NAFLD patients (58.1% vs. 48.9%, p < 0.001). Non-obese women with fibrosis had the highest FPG and HbA1c levels and the lowest HDL-C levels, but the differences were not significant. They were most likely to have poor TG control and had higher prevalence of HFpEF (Supplementary Tables 2–5). Estimates across subgroups should be interpreted with caution because of limited sample size and inadequate statistical power.

### Sensitivity analyses

When a more stringent BMI cutoff of 23 kg/m^2^ was used to classify patients as lean and non-lean, results were essentially the same (Supplementary Tables 6–8).

After excluding patients taking TZDs, SGLT-2Is, and/or GLP-1RAs, which may have effects on weight and liver fat content [22], results were essentially the same (Supplementary Tables 9 and 10). To rule out the impact of T2DM duration on the results, we conducted subgroup analyses in patients with T2DM duration ≥ and < 5 years, and the results were also essentially the same in these two subgroups (Supplementary Tables 11–14).

## Discussion

This is, as far as we are aware, the first report to describe the metabolic and cardiovascular risk profile of non-obese NAFLD subjects in comparison with obese subjects without NAFLD and with NAFLD in type 2 diabetic patients. We found that in patients with T2DM, non-obese NAFLD subjects had more and worse metabolic disorders than obese subjects without NAFLD among both men and women. Comparable cardio-metabolic risk profiles were noted between non-obese and obese NAFLD patients. Among women with T2DM, the associations of worse cardio-metabolic risk profiles with NAFLD were overall stronger in non-obese patients than in obese patients. Among men with T2DM, however, the associations were more pronounced in obese than in non-obese patients.

The non-obese NAFLD phenotype has sparked interest because of its high prevalence [5], unanswered questions regarding its pathophysiological mechanisms, and whether stratifying NAFLD patients based on their obesity status could prioritize allocation of clinical resources for those most at risk of poor outcomes [5]. Wei et al. reported that one-fifth of non-obese Chinese had magnetic resonance spectroscopy-defined NAFLD [23]. Conflicting results have been observed regarding cardio-metabolic risk profiles in non-obese NAFLD subjects: Semmler et al. evidenced that non-obese NAFLD was associated with metabolic disorders intermediate between non-obese controls and obese NAFLD [8]. That is, non-obese NAFLD subjects generally had a milder metabolic phenotype compared with NAFLD subjects who were additionally obese, while more and worse metabolic derangements than non-obese controls. Other reports convinced that non-obese NAFLD subjects had severe impaired glucose tolerance and dyslipidemia that were identical or even worse than obese NAFLD subjects [6, 9, 24]. These existing broad evidence from general population-based analyses supports that non-obese NAFLD may represent a distinct entity in the disease spectrum of NAFLD. To date, a comprehensive analysis of cardio-metabolic characteristics in non-obese NAFLD subjects has not been reported in type 2 diabetic patients, in whom non-obese and obese NAFLD were both more frequent.

We addressed this fundamental knowledge gap in the present study. We found that in T2DM, non-obese NAFLD subjects had more and worse metabolic abnormalities than obese subjects without NAFLD. Some prospective cohort studies showed that lean NAFLD participants were at a higher risk of incident diabetes than obese participants without NAFLD [25, 26]. Our results together with findings from the above reports suggest that NAFLD was a much stronger indicator of metabolic disorders than obesity. Furthermore, our study also suggested that NAFLD in type 2 diabetic subjects, even if they were not obese, might be better identified as an indicator of the presence of poor achievements of the ADA guideline-recommended HbA1c, BP, and lipid levels. One possible explanation for this result may be due to a decreased capacity for storing fat in adipose tissue in non-obese NAFLD patients. Mice impairing fat-storage ability in adipocytes showed severe non-obese NAFLD under high fat diet circumstances [27]. Non-obese NAFLD may have a lipodystrophy-like phenotype, characterized by impaired adipogenesis, hypertriglyceridemia, and hepatic steatosis [28]. According to the overflow hypothesis, adipose tissue acts as a reservoir of free fatty acids and prevents their overflow into insulin-sensitive tissues including liver. Alterations in fatty acid trafficking leads to abnormalities in lipid storage and consequent ectopic fat deposition [29]. Further studies to examine the potential mechanisms for non-obese NAFLD are warranted.

In the present study, in order to avoid treatment bias for BP, glucose, and lipid values, we showed the anti-diabetic drugs-, anti-hypertensive drugs-, and lipid-lowering drugs-adjusted means. We found that in patients with T2DM, non-obese NAFLD patients share many cardio-metabolic disorders with obese NAFLD patients, with similar levels of BP, FPG, HbA1c, and lipid profiles in non-obese and obese NAFLD patients. In previous studies conducted in the general population, Kwon et al. found that non-obese NAFLD individuals had milder metabolic derangements compared with obese NAFLD subjects [7]. Two studies reported that non-obese NAFLD subjects had comparable LDL-C levels, while milder profiles of other components of metabolic syndrome than obese NAFLD counterparts [6, 8]. A recent meta-analysis showed that FPG, 2-hour postprandial glucose, TC, and LDL-C were similar in non-obese and obese NAFLD patients [9]. Our results together with findings from the above reports suggest that individuals who develop NAFLD have less metabolic adaptability at a given weight gain compared with obese NAFLD patients. The exact mechanisms for this difference remain unclear. Evidence showed that patients with non-obese NAFLD differed from obese NAFLD patients in genetic predisposition [30]. Several genotypes, such as PNPLA3 and TM6SF2, which are strongly associated with hepatic fat content, were found to have a predominant role in non-obese NAFLD subjects [30]. One cohort study conducted in Chinese population showed that lean NAFLD individuals had a higher visceral adiposity index than overweight/obese NAFLD subjects [31], while in our study, non-obese and obese NAFLD patients have similar visceral adiposity, as evidenced by comparable visceral to subcutaneous adipose tissue ratio. Besides, the Rotterdam study indicates that skeletal muscle mass was consistently associated with NAFLD in normal-weight women [32]. Hence, increased visceral fat with or without sarcopenia also contributes to the less metabolic adaptability in non-obese NAFLD. Further, the complex interplay among multiple factors including genetics, diets, lifestyles, and gut microbiota is likely to modify individuals’ metabolic adaptation [33].

Our data revealed that in patients with T2DM, NAFLD patients were less likely to have the care goal achievement (especially for dyslipidemia and dysglycemia), regardless of obesity status. However, the associations were stronger in non-obese patients than in obese patients among women, while among men, the presence of poor control of HbA1c and lipids were more strongly associated with NAFLD in obese patients than in non-obese patients. Previous study conducted in health examination population also showed that differences in odds of NAFLD for components of metabolic syndrome between non-obese and obese subjects were more significant in women than in men [7]. There is a well-recognized disparity in cardio-metabolic risk profile by sex among individuals with diabetes, with stronger effect of diabetes on cardio-metabolic risk profiles in women as compared to men [34].

The main strength of this study is the large number of T2DM patients included from an academic hospital. Further, we can get access to clinical, laboratory, and imaging data in medical records, which provided more in-depth clinical information that are not usually available in large epidemiological surveys.

The limitations of our study are as follows. First of all, our study population were mainly based on inpatients suffering from T2DM, whose health conditions might be severer than those of outpatients. Thus, our findings could not be generalized to outpatients with T2DM. Second, NAFLD was diagnosed by ultrasonography after exclusion of secondary causes for steatosis. Although ultrasonography is widely used to define fatty liver, operator-dependency, false-negativity, and inability to quantify liver fat are major limitations of this test [35]. Third, severity of steatosis was not defined for all patients. Fourth, information on dietary habits, which might be different between obese and non-obese NAFLD patients [36], is lacking. Finally, BMI was used to define obesity status, while the limitation of BMI as an adiposity indicator is well recognized [37].

## Conclusion

Non-obese NAFLD subjects with T2DM can demonstrate the full spectrum of metabolic disorders that occurs in obese NAFLD patients. Our findings suggest that non-obese NAFLD patients with T2DM require careful monitoring for the presence and development of metabolic abnormalities. Future research is needed to provide a better understanding of the mechanisms for the development of non-obese NAFLD in patients with T2DM, and its long-term clinical implications.

## Electronic supplementary material

Below is the link to the electronic supplementary material.

Supplementary Material 1

## Data Availability

The datasets used and/or analyzed during the current study are available from the corresponding author on reasonable request.

## Abbreviations

NAFLD: Non-alcoholic fatty liver disease.
T2DM: Type 2 diabetes mellitus.
BMI: Body mass index.
BP: blood pressure.
FPG: Fasting plasma glucose.
HOMA-IR: Homeostasis model assessment of insulin resistance.
HbA1c: Glycated hemoglobin.
TC: Total cholesterol.
TG: Triglycerides.
LDL-C: Low density lipoprotein cholesterol.
HDL-C: High density lipoprotein cholesterol.
ALT: Alanine aminotransferase; AST, aspartate aminotransferase.
γ-GT: γ-Glutamyl transferase.
Cr: Creatinine.
UA: Uric acid.
eGFR: Estimated glomerular filtration rate.
UACR: Urinary albumin to creatinine ratio.
HFpEF: Heart failure with preserved ejection fraction.
LVH: Left ventricular hypertrophy.
FIB-4: Index, Fibrosis-4 index.
NFS: NAFLD Fibrosis Score.
IMT: Intima-media thickness.
SAT: Subcutaneous adipose tissue.
VAT: Visceral adipose tissue.
VSR: Visceral to subcutaneous adipose tissue ratio.
SUs: Sulfonylureas.
AGIs: α-glucosidase inhibitors.
TZDs: Thiazolidinediones.
DPP-4Is: Dipeptidyl peptidase-4 inhibitors.
SGLT-2Is: Sodium-glucose cotransporter-2 inhibitors.
GLP-1RAs: Glucagon-like peptide-1 receptor agonists.
CVD: Cardiovascular disease.
