# Supplementary material for: Non-obese NAFLD had no better cardio-metabolic risk profile than obese NAFLD in type 2 diabetic patients
Source: Cardiovasc Diabetol. 2022 Oct 14;21:210. doi: 10.1186/s12933-022-01648-9 (PMC9569122; doi:10.1186/s12933-022-01648-9)
Supplement: Supplementary file 1 — Supplementary Material 1 [file 12933_2022_1648_MOESM1_ESM.docx]

Supplementary Table 1. Use of anti-diabetic drugs in the study population

|  | n (%) |
| --- | --- |
| SUs | 475 (17.5) |
| Non-SUs | 69 (2.55) |
| Biguanides | 895 (33.1) |
| AGIs | 659 (24.3) |
| TZDs | 193 (7.13) |
| DPP-4Is | 235 (8.68) |
| SGLT-2Is | 115 (4.25) |
| Insulin | 849 (31.4) |
| GLP-1RAs | 39 (1.44) |

SUs, sulfonylureas; AGIs, α-glucosidase inhibitors; TZDs, thiazolidinediones; DPP-4Is, dipeptidyl peptidase-4 inhibitors; SGLT-2Is, sodium-glucose cotransporter-2 inhibitors; GLP-1RAs, glucagon-like peptide-1 receptor agonists

Supplementary Table 2. Adjusted means of cardio-metabolic risk factors according to obesity and fibrosis status defined by FIB-4 index in NAFLD patients^a^

|  | Without fibrosis | | With fibrosis | |
| --- | --- | --- | --- | --- |
|  | Obese | Non-obese | Obese | Non-obese |
| **Women** | n = 176 | n = 146 | n = 68 | n = 55 |
| Systolic BP (mmHg) | 138 (132, 145) | 136 (130, 142) | 138 (130, 145) | 139 (130, 147) |
| Diastolic BP (mmHg) | 82.6 (78.8, 86.4) | 80.5 (76.9, 84.2) | 79.9 (75.4, 84.5) | 83.2 (78.4, 88.0) |
| FPG (mmol/L) | 9.34 (7.91, 10.8) | 9.42 (8.10, 10.7) | 9.21 (7.42, 11.0) | 10.44 (8.61, 12.3) |
| HbA1c (%) | 8.92 (8.18, 9.65) | 9.43 (8.72, 10.13) | 9.28 (8.40, 10.16) | 9.89 (8.96, 10.83) |
| TC (mg/dL) | 176 (160, 192) | 181 (166, 196) | 169 (150, 188) | 174 (153, 194) |
| TG (mg/dL) | 301 (231, 370) | 355 (288, 421) | 296 (213, 379) | 353 (265, 442) |
| LDL-C (mg/dL) | 102.4 (90.4, 114) | 99.1 (87.6, 111) | 94.0 (79.7, 108) | 91.8 (76.6, 107) |
| HDL-C (mg/dL) | 41.8 (38.8, 44.8) | 42.5 (39.6, 45.4) | 40.8 (37.2, 44.4) | 38.9 (35.1, 42.7) |
| Non-HDL-C (mg/dL) | 134 (119, 149) | 139 (124, 153) | 128 (111, 146) | 132 (113, 151) |
| **Men** | n = 479 | n = 217 | n = 115 | n = `61 |
| Systolic BP (mmHg) | 136 (130, 141) | 130 (122, 137) | 135 (126, 144) | 132 (120, 144) |
| Diastolic BP (mmHg) | 86.4 (84.9, 87.9) | 84.6 (82.5, 86.7) | 85.5 (82.9, 88.0) | 87.8 (84.4, 91.1) |
| FPG (mmol/L) | 9.32 (8.66, 9.97) | 8.71 (7.80, 9.62) | 9.59 (8.41, 10.78) | 8.36 (6.95, 9.77) |
| HbA1c (%) | 9.50 (9.22, 9.78) | 9.80 (9.41, 10.19) | 9.46 (8.99, 9.94) | 9.42 (8.79, 10.05) |
| TC (mg/dL) | 180 (174, 186) | 173 (164, 181) | 173 (163, 184) | 174 (160, 188) |
| TG (mg/dL) | 380 (340, 420) | 353 (296, 409) | 352 (284, 420) | 351 (261, 440) |
| LDL-C (mg/dL) | 99.9 (95.3, 104) | 101.4 (95.1, 108) | 94.2 (86.5, 102) | 97.3 (87.1, 107) |
| HDL-C (mg/dL) | 34.0 (33.1, 35.0) | 35.2 (33.8, 36.5) | 36.0 (34.4, 37.6) | 34.5 (32.3, 36.6) |
| Non-HDL-C (mg/dL) | 146 (140, 152) | 137 (128, 145) | 137 (127, 148) | 139 (125, 152) |

FIB-4 index, Fibrosis-4 index; BP, blood pressure; FPG: fasting plasma glucose; HbA1c, glycated hemoglobin; TC, total cholesterol; TG, triglycerides; LDL-C, low density lipoprotein cholesterol; HDL-C, high density lipoprotein cholesterol.

Data are presented as means (95% confidence intervals)

^a^ Data were adjusted for age, waist circumference, smoking status, anti-diabetic drugs, anti-hypertensive drugs, and lipid-lowering drugs.

Supplementary Table 3. Associations of fibrosis status defined by FIB-4 index with cardio-metabolic risk profiles according to obesity status in NAFLD patients ^a^

|  | | Crude OR | | | | Adjusted OR ^a^ | | |
| --- | --- | --- | --- | --- | --- | --- | --- | --- |
|  | Non-obese w/o fibrosis | | Obese with fibrosis | Non-obese with fibrosis | Non-obese w/o fibrosis | | Obese with fibrosis | Non-obese with fibrosis |
| **Women** |  | |  |  |  | |  |  |
| BP ≥ 130/80 mmHg | 0.44 (0.27,0.72) | | 0.69 (0.36,1.3) | 0.71 (0.35,1.42) | 0.86 (0.46, 1.63) | | 0.95 (0.45, 1.99) | 1.36 (0.57, 3.27) |
| HbA1c ≥ 7.0% | 1.06 (0.53,2.13) | | 1.13 (0.45,2.81) | 1.61 (0.53,4.94) | 1.01 (0.41, 2.50) | | 0.97 (0.35, 2.65) | 1.53 (0.40, 5.80) |
| TG ≥ 150mg/dL | 1.12 (0.69,1.82) | | 1.55 (0.8,2.99) | 1.01 (0.52,1.98) | 1.68 (0.89, 3.15) | | 1.65 (0.80, 3.41) | 1.60 (0.69, 3.72) |
| LDL-C ≥ 100mg/dL | 0.89 (0.56,1.41) | | 0.79 (0.44,1.42) | 0.55 (0.29,1.03) | 0.72 (0.39, 1.35) | | 0.71 (0.36, 1.41) | 0.52 (0.23, 1.19) |
| HDL-C ≤ 40/50 mg/dL for men/women | 0.46 (0.25,0.83) | | 0.72 (0.32,1.58) | 0.78 (0.32,1.87) | 0.63 (0.30, 1.31) | | 0.85 (0.36, 2.01) | 1.07 (0.38, 2.96) |
| HFpEF | 1.86 (1.07,3.24) | | 0.99 (0.46,2.12) | 0.92 (0.39,2.15) | 1.87 (0.90, 3.87) | | 1.27 (0.55, 2.94) | 0.90 (0.30, 2.73) |
| LVH | 0.85 (0.5,1.43) | | 1.16 (0.62,2.19) | 0.8 (0.38,1.7) | 0.82 (0.40, 1.65) | | 1.01 (0.49, 2.07) | 0.62 (0.24, 1.59) |
| **Men** |  | |  |  |  | |  |  |
| BP ≥ 130/80 mmHg | 0.59 (0.41,0.85) | | 0.84 (0.52,1.36) | 0.79 (0.42,1.47) | 0.70 (0.45, 1.09) | | 0.78 (0.46, 1.32) | 0.79 (0.40, 1.55) |
| HbA1c ≥ 7.0% | 1.00 (0.60, 1.67) | | 0.63 (0.36,1.11) | 0.57 (0.28,1.16) | 0.86 (0.47, 1.59) | | 0.64 (0.34, 1.20) | 0.49 (0.22, 1.01) |
| TG ≥ 150 mg/dL | 0.61 (0.42,0.9) | | 0.74 (0.45,1.2) | 0.62 (0.33,1.15) | 0.71 (0.45, 1.12) | | 0.84 (0.49, 1.43) | 0.79 (0.40, 1.55) |
| LDL-C ≥ 100 mg/dL | 1.23 (0.89,1.71) | | 0.79 (0.52,1.19) | 1.13 (0.65,1.95) | 0.96 (0.65, 1.41) | | 0.84 (0.53, 1.34) | 1.03 (0.56, 1.91) |
| HDL-C ≤ 40/50 mg/dL for men/women | 0.53 (0.36,0.76) | | 0.44 (0.28,0.69) | 0.35 (0.2,0.63) | 0.64 (0.41, 1.00) | | 0.54 (0.33, 0.89) | 0.52 (0.27, 0.97) |
| HFpEF | 1.25 (0.84,1.84) | | 1.35 (0.82,2.21) | 1.02 (0.52,2.01) | 1.31 (0.81, 2.12) | | 1.05 (0.61, 1.81) | 0.88 (0.41, 1.85) |
| LVH | 0.45 (0.18,1.11) | | 1.52 (0.72,3.23) | 0.54 (0.13,2.32) | 0.38 (0.14, 1.08) | | 0.74 (0.32, 1.73) | 0.32 (0.07, 1.49) |

FIB-4 index, Fibrosis-4 index; BP, blood pressure; HbA1c, glycated hemoglobin; TG, triglycerides; LDL-C, low density lipoprotein cholesterol; HDL-C, high density lipoprotein cholesterol; HFpEF, heart failure with preserved ejection fraction; LVH, left ventricular hypertrophy.

Data are presented as odds ratios (95% confidence intervals).

^a^ Models were adjusted for age, waist circumference, smoking status, HbA1c, BP, LDL-C, duration of T2DM, anti-diabetic drugs, anti-hypertensive drugs, and lipid-lowering drugs.

Supplementary Table 4. Adjusted means of cardio-metabolic risk factors according to obesity and fibrosis status defined by NFS in NAFLD patients^a^

|  | Without fibrosis | | With fibrosis | |
| --- | --- | --- | --- | --- |
|  | Obese | Non-obese | Obese | Non-obese |
| **Women** | n = 97 | n = 96 | n = 135 | n = 97 |
| Systolic BP (mmHg) | 138 (130, 145) | 137 (130, 144) | 136 (129, 143) | 136 (129, 143) |
| Diastolic BP (mmHg) | 83.1 (78.6, 87.6) | 80.9 (76.7, 85.1) | 81.5 (77.3, 85.7) | 82.8 (78.6, 87.1) |
| FPG (mmol/L) | 9.52 (7.80, 11.2) | 9.93 (8.32, 11.5) | 9.19 (7.43, 11.0) | 9.68 (8.03, 11.3) |
| HbA1c (%) | 8.28 (7.43, 9.12) | 9.25 (8.45, 10.5) ^*^ | 9.25 (8.46, 10.05) ^*^ | 9.65 (8.85, 10.44) ^*^ |
| TC (mg/dL) | 186 (167, 205) | 188 (170, 206) | 175 (158, 193) | 182 (165, 200) |
| TG (mg/dL) | 323 (239, 407) | 381 (303, 460) | 320 (243, 398) | 372 (294, 449) |
| LDL-C (mg/dL) | 109.7 (95.6, 124) | 105.1 (91.9, 118) | 97.6 (84.5, 111) | 95.0 (81.9, 108) |
| HDL-C (mg/dL) | 41.9 (38.4, 45.5) | 43.0 (39.7, 46.3) | 40.9 (37.6, 44.2) | 40.0 (36.8, 43.3) |
| Non-HDL-C (mg/dL) | 143 (126, 161) | 146 (129, 162) | 135 (118, 151) | 141 (124, 157) |
| **Men** | n = 237 | n = 154 | n = 329 | n = `115 |
| Systolic BP (mmHg) | 133 (125, 140) | 129 (120, 137) | 138 (132, 144) | 132 (123, 142) |
| Diastolic BP (mmHg) | 87.1 (85.2, 89.0) | 84.0 (81.7, 86.3) | 85.7 (84.0, 87.4) | 87.5 (84.8, 90.1) |
| FPG (mmol/L) | 9.41 (8.56, 10.26) | 8.54 (7.55, 9.53) | 9.42 (8.64, 10.20) | 8.56 (7.43, 9.68) |
| HbA1c (%) | 9.50 (9.14, 9.86) | 9.70 (9.26, 10.14) | 9.48 (9.16, 9.80) | 9.78 (9.30, 10.27) |
| TC (mg/dL) | 183 (175, 191) | 174 (164, 184) | 177 (170, 184) | 171 (160, 181) |
| TG (mg/dL) | 363 (311, 415) | 329 (265, 392) | 389 (342, 435) | 378 (307, 448) |
| LDL-C (mg/dL) | 105.3 (99.5, 111.1) | 105.2 (98.2, 112.2) | 94.3 (89.2, 99.4) ^*†^ | 93.7 (86.0, 101.5) |
| HDL-C (mg/dL) | 34.6 (33.3, 35.8) | 35.7 (34.2, 37.2) | 34.0 (32.9, 35.1) | 33.9 (32.3, 35.6) |
| Non-HDL-C (mg/dL) | 148 (140, 155) | 137 (128, 147) | 143 (136, 150) | 136 (125, 147) |

NFS, NAFLD Fibrosis Score; BP, blood pressure; FPG: fasting plasma glucose; HbA1c, glycated hemoglobin; TC, total cholesterol; TG, triglycerides; LDL-C, low density lipoprotein cholesterol; HDL-C, high density lipoprotein cholesterol.

Data are presented as means (95% confidence intervals)

^a^ Data were adjusted for age, waist circumference, smoking status, anti-diabetic drugs, anti-hypertensive drugs, and lipid-lowering drugs.

^*^ p < 0.05 compared with the group of obese NAFLD patients without fibrosis;

^†^ p < 0.05 compared with the group of non-obese NAFLD patients without fibrosis.

Supplementary Table 5. Associations of fibrosis status defined by NFS with cardio-metabolic risk profiles according to obesity status in NAFLD patients ^a^

|  | | Crude OR | | | | Adjusted OR ^a^ | | |
| --- | --- | --- | --- | --- | --- | --- | --- | --- |
|  | Non-obese w/o fibrosis | | Obese with fibrosis | Non-obese with fibrosis | Non-obese w/o fibrosis | | Obese with fibrosis | Non-obese with fibrosis |
| **Women** |  | |  |  |  | |  |  |
| BP ≥ 130/80 mmHg | 0.38 (0.2,0.73) | | 0.63 (0.33,1.19) | 0.49 (0.25,0.94) | 0.64 (0.29, 1.42) | | 0.54 (0.26, 1.13) | 0.75 (0.32, 1.74) |
| HbA1c ≥ 7.0% | 2.06 (0.83,5.13) | | 2.11 (0.92,4.82) | 1.61 (0.69,3.8) | 2.18 (0.71, 6.71) | | 1.77 (0.70, 4.48) | 1.45 (0.48, 4.40) |
| TG ≥ 150mg/dL | 1.11 (0.59,2.06) | | 1.18 (0.66,2.1) | 1.11 (0.59,2.06) | 1.39 (0.65, 2.96) | | 1.00 (0.52, 1.91) | 1.55 (0.70, 3.44) |
| LDL-C ≥ 100mg/dL | 0.7 (0.37,1.31) | | 0.45 (0.25,0.8) | 0.41 (0.22,0.76) | 0.63 (0.30, 1.36) | | 0.56 (0.29, 1.07) | 0.49 (0.22, 1.06) |
| HDL-C ≤ 40/50 mg/dL for men/women | 0.51 (0.25,1.06) | | 1.26 (0.58,2.72) | 0.92 (0.42,2.03) | 0.71 (0.30, 1.70) | | 1.32 (0.58, 3.02) | 1.21 (0.47, 3.11) |
| HFpEF | 1.99 (0.95,4.17) | | 1.32 (0.65,2.7) | 1.64 (0.78,3.47) | 2.09 (0.84, 5.22) | | 1.76 (0.78, 3.95) | 2.22 (0.84, 5.84) |
| LVH | 1.60 (0.77,3.35) | | 2.32 (1.2,4.51) | 1.32 (0.62,2.78) | 1.58 (0.64, 3.91) | | 1.95 (0.90, 4.20) | 0.97 (0.38, 2.49) |
| **Men** |  | |  |  |  | |  |  |
| BP ≥ 130/80 mmHg | 0.58 (0.36,0.94) | | 0.82 (0.54,1.23) | 0.55 (0.33,0.92) | 0.67 (0.39, 1.14) | | 0.80 (0.50, 1.30) | 0.66 (0.36, 1.20) |
| HbA1c ≥ 7.0% | 0.66 (0.33,1.33) | | 0.43 (0.25,0.76) | 0.52 (0.25,1.05) | 0.63 (0.29, 1.40) | | 0.52 (0.27, 0.99) | 0.50 (0.22, 1.16) |
| TG ≥ 150 mg/dL | 0.61 (0.37,0.99) | | 0.87 (0.57,1.34) | 0.55 (0.33,0.94) | 0.76 (0.43, 1.33) | | 1.01 (0.62, 1.66) | 0.76 (0.41, 1.41) |
| LDL-C ≥ 100 mg/dL | 1.16 (0.76,1.77) | | 0.56 (0.4,0.79) | 0.68 (0.43,1.06) | 0.97 (0.60, 1.58) | | 0.61 (0.41, 0.91) | 0.55 (0.32, 0.94) |
| HDL-C ≤ 40/50 mg/dL for men/women | 0.49 (0.3,0.79) | | 0.76 (0.5,1.16) | 0.45 (0.27,0.76) | 0.65 (0.38, 1.11) | | 1.09 (0.67, 1.76) | 0.75 (0.41, 1.35) |
| HFpEF | 1.48 (0.85,2.57) | | 2.33 (1.48,3.65) | 2.66 (1.54,4.62) | 1.32 (0.70, 2.47) | | 1.86 (1.11, 3.10) | 2.22 (1.17, 4.22) |
| LVH | 0.75 (0.22,2.54) | | 2.55 (1.13,5.71) | 1.02 (0.3,3.46) | 0.45 (0.12, 1.71) | | 1.12 (0.46, 2.74) | 0.47 (0.12, 1.75) |

NFS, NAFLD Fibrosis Score; BP, blood pressure; HbA1c, glycated hemoglobin; TG, triglycerides; LDL-C, low density lipoprotein cholesterol; HDL-C, high density lipoprotein cholesterol; HFpEF, heart failure with preserved ejection fraction; LVH, left ventricular hypertrophy.

Data are presented as odds ratios (95% confidence intervals).

^a^ Models were adjusted for age, waist circumference, smoking status, HbA1c, BP, LDL-C, duration of T2DM, anti-diabetic drugs, anti-hypertensive drugs, and lipid-lowering drugs.

Supplementary Table 6. Characteristics of the study population (n = 2708) according to weight (23 kg/m^2^) and NAFLD status

|  | Without NAFLD | | NAFLD | |
| --- | --- | --- | --- | --- |
|  | Lean  (n = 578) | Non-lean  (n = 795) | Non-lean  (n = 1142) | Lean  (n = 193) |
| Men (%) | 299 (51.7) | 516 (64.9) ^*^ | 790 (69.2) ^*^ | 96 (49.7) ^†§^ |
| Age (year) | 54.1 (12.4) | 56.2 (11.8) ^*^ | 49.9 (13.4) ^*†^ | 52.4 (11.8) ^†^ |
| BMI (kg/m^2^) | 20.8 (1.62) | 25.9 (2.46) ^*^ | 27.3 (3.21) ^*†^ | 21.6 (1.45) ^*†§^ |
| Waist circumstance (cm) | 84.2 (33.0) | 94.4 (8.07) ^*^ | 97.7 (9.37) ^*†^ | 86.3 (6.31) ^†§^ |
| Systolic BP (mmHg) | 127 (20.4) | 134 (21.1) ^*^ | 134 (37.4) ^*^ | 128 (19.5) ^†§^ |
| Diastolic BP (mmHg) | 79.2 (11.9) | 81.0 (12.3) ^*^ | 85.0 (12.2) ^*^ | 81.1 (11.9) ^§^ |
| FPG (mmol/L) | 8.57 (3.45) | 8.23 (2.84) | 9.07 (3.44) ^†^ | 9.28 (3.55) ^†^ |
| HOMA-IR | 1.58 (0.80–2.83) | 2.18 (1.10–4.57) | 3.04 (1.53–5.08) | 2.28 (1.35–4.27) ^*§^ |
| HbA1c (%) | 9.28 (2.62) | 8.87 (2.35) ^*^ | 9.55 (2.23) ^†^ | 9.95 (2.56) ^*†^ |
| TC (mg/dL) | 171 (46.6) | 167 (44.2) | 183 (50.3) ^*†^ | 187 (53.2) ^*†^ |
| TG (mg/dL) | 131 (87.7–210) | 159 (107–242) ^*^ | 237 (155–381) ^*†^ | 209 (146–415) ^*†^ |
| LDL-C (mg/dL) | 104 (36.4) | 103 (37.1) | 108 (36.9) ^†^ | 109 (39.1) |
| HDL-C (mg/dL) | 44.9 (13.2) | 40.9 (10.7) ^*^ | 36.9 (8.34) ^*†^ | 38.7 (10.5) ^*†^ |
| Non-HDL-C (mg/dL) | 126 (44.7) | 126 (42.5) | 146 (50.1) ^*†^ | 147 (49.7) ^*†^ |
| ALT (U/L) | 15.0 (11.0–23.0) | 17.0 (13.0–25.0) ^*^ | 25.0 (18.0–38.0) ^*†^ | 20.0 (14.0–28.0) ^*†§^ |
| AST (U/L) | 17.0 (14.0–21.0) | 18.0 (14.0–22.0) ^*^ | 20.0 (16.0–28.0) ^*†^ | 18.0 (14.0–22.0) ^*§^ |
| γ-GT (U/L) | 20.0 (14.0–30.0) | 23.0 (17.0–35.0) ^*^ | 36.0 (24.0–54.0) ^*†^ | 30.5 (19.0–45.0) ^*†§^ |
| sCr (μmol/L) | 66.0 (54.0–83.0) | 74.0 (60.0–92.0) ^*^ | 70.0 (59.0–82.0) ^*†^ | 61.5 (52.0–77.0) ^*†§^ |
| sUA (μmol/L) | 291 (95.2) | 333 (203) ^*^ | 357 (102) ^*†^ | 310 (95.9) ^§^ |
| eGFR (mL/min/1.73 m^2^) | 93.1 (26.2) | 86.3 (26.1) ^*^ | 98.2 (21.9) ^*†^ | 98.8 (23.3) ^*†^ |
| UACR (mg/g) | 15.0 (6.90–47.0) | 15.1 (7.00–72.4) | 14.4 (6.60–44.9) | 12.4 (7.30–26.5) |
| T2DM duration (year) | 7.48 (7.05) | 8.42 (7.43) | 5.05 (6.13) ^*†^ | 5.65 (6.63) ^*†^ |
| Smoking (%) | 134 (23.2) | 222 (27.9) | 372 (32.6) ^*^ | 43 (22.3) ^§^ |
| BP ≥ 130/80 mmHg (%) | 334 (57.8) | 544 (68.4) ^*^ | 871 (76.3) ^*†^ | 123 (63.7) ^§^ |
| HbA1c ≥ 7.0% (%) | 443 (78.3) | 606 (77.3) | 996 (88.4) ^*†^ | 169 (88.0) ^*†^ |
| TG ≥ 150mg/dL (%) | 243 (42.6) | 421 (53.7) ^*^ | 868 (76.4) ^*†^ | 141 (74.6) ^*†^ |
| LDL-C ≥ 100mg/dL (%) | 290 (50.9) | 406 (51.8) | 657 (58.2) ^*†^ | 109 (57.7) |
| HDL-C ≤ 40/50 mg/dL for men/women (%) | 319 (56.0) | 498 (63.4) ^*^ | 884 (78.3) ^*†^ | 142 (75.1) ^*†^ |
| HFpEF (%) | 88 (16.4) | 134 (18.1) | 227 (21.0) | 42 (23.6) |
| LVH (%) | 69 (12.5) | 139 (18.3) ^*^ | 125 (11.3) ^†^ | 27 (14.9) |
| Anti-diabetic drugs (%) | 423 (73.2) | 545 (68.6) | 683 (59.8) ^*†^ | 116 (60.1) ^*†^ |
| Anti-hypertensive drugs (%) | 150 (26.0) | 354 (44.5) ^*^ | 432 (37.8) ^*†^ | 51 (26.4) ^†§^ |
| Lipid-lowering drugs (%) | 57 (9.86) | 117 (14.7) | 143 (12.5) | 20 (10.4) |

BMI, body mass index; BP, blood pressure; FPG: fasting plasma glucose; HOMA-IR, homeostasis model assessment of insulin resistance; HbA1c, glycated hemoglobin; TC, total cholesterol; TG, triglycerides; LDL-C, low density lipoprotein cholesterol; HDL-C, high density lipoprotein cholesterol; ALT, alanine aminotransferase; AST, aspartate aminotransferase; γ-GT, γ-glutamyl transferase; Cr, creatinine; UA, uric acid; eGFR, estimated glomerular filtration rate; UACR, urinary albumin to creatinine ratio; T2DM, type 2 diabetes mellitus; HFpEF, heart failure with preserved ejection fraction; LVH, left ventricular hypertrophy.

Data are presented as means (SDs), medians (IQRs), or numbers (percentages) depending on their distribution.

^*^ p < 0.05 compared with the group of lean without NAFLD;

^†^ p < 0.05 compared with the group of non-lean without NAFLD;

^§^ p < 0.05 compared with the group of non-lean NAFLD.

Supplementary Table 7. Adjusted means of cardio-metabolic risk factors according to weight (23 kg/m^2^) and NAFLD status in women and men ^a^

|  | Without NAFLD | | NAFLD | |
| --- | --- | --- | --- | --- |
|  | Lean | Non-lean | Non-lean | Lean |
| **Women** | n = 279 | n = 279 | n = 351 | n = 97 |
| Systolic BP (mmHg) | 132 (127, 137) | 134 (129, 139) | 133 (129, 138) | 133 (127, 139) |
| Diastolic BP (mmHg) | 77.0 (74.0, 80.0) | 77.5 (74.6, 80.4) | 80.3 (77.5, 83.1) ^*†^ | 78.3 (74.9, 81.7) |
| FPG (mmol/L) | 9.12 (8.10, 10.1) | 8.37 (7.42, 9.33) | 9.00 (8.08, 9.92) | 10.1 (8.89, 11.2) ^†^ |
| HbA1c (%) | 9.11 (8.52, 9.71) | 8.58 (8.02, 9.15) | 9.45 (8.90, 10.0) ^†^ | 9.77 (9.10, 10.4) ^†^ |
| TC (mg/dL) | 160 (148, 172) | 156 (145, 168) | 171 (160, 182) ^†^ | 173 (159, 187) ^†^ |
| TG (mg/dL) | 222 (176, 268) | 210 (166, 254) | 284 (242, 327) ^*†^ | 297 (245, 349) ^*†^ |
| LDL-C (mg/dL) | 93.8 (84.3, 103) | 89.8 (80.7, 98.8) | 99.0 (90.1, 108) ^†^ | 98.3 (87.5, 109) |
| HDL-C (mg/dL) | 45.5 (42.6, 48.4) | 43.7 (40.9, 46.5) | 41.5 (38.8, 44.2) ^*^ | 40.0 (36.7, 43.3) ^*^ |
| Non-HDL-C (mg/dL) | 115 (104, 127) | 113 (102, 124) | 130 (120, 141) ^*†^ | 132 (119, 145) ^*†^ |
| **Men** | n = 299 | n = 516 | n = 790 | n = 96 |
| Systolic BP (mmHg) | 127 (122, 131) | 133 (129, 136) | 133 (130, 137) ^*^ | 127 (119, 134) |
| Diastolic BP (mmHg) | 80.4 (78.7, 82.0) | 81.9 (80.7, 83.2) | 84.5 (83.4, 85.6) ^*†^ | 82.9 (80.3, 85.5) |
| FPG (mmol/L) | 8.67 (7.96, 9.38) | 8.44 (7.94, 8.95) | 9.17 (8.71, 9.63) | 8.89 (7.81, 9.97) |
| HbA1c (%) | 9. 46 (9.14, 9.78) | 9.19 (8.95, 9.43) | 9.40 (9.19, 9.61) | 9.65 (9.14, 10.2) |
| TC (mg/dL) | 158 (151, 164) | 162 (157, 167) | 174 (170, 178) ^*†^ | 176 (166, 187) ^*†^ |
| TG (mg/dL) | 214 (178, 250) | 246 (218, 274) | 345 (321, 369) ^*†^ | 396 (338, 454) ^*†^ |
| LDL-C (mg/dL) | 92.2 (87.2, 97.1) | 97.9 (94.2, 102) | 98.7 (95.4, 102) | 96.8 (88.9, 105) |
| HDL-C (mg/dL) | 40.6 (39.3, 41.9) | 37.8 (36.8, 38.7) ^*^ | 35.1 (34.3, 36.0) ^*†^ | 35.0 (33.0, 37.0) ^*†^ |
| Non-HDL-C (mg/dL) | 117 (111, 123) | 124 (120, 129) | 138 (134, 143) ^*†^ | 140 (130, 151) ^*†^ |

BP, blood pressure; FPG: fasting plasma glucose; HbA1c, glycated hemoglobin; TC, total cholesterol; TG, triglycerides; LDL-C, low density lipoprotein cholesterol; HDL-C, high density lipoprotein cholesterol.

Data are presented as means (95% confidence intervals).

^a^ Data were adjusted for age, waist circumference, smoking status, anti-diabetic drugs, anti-hypertensive drugs, and lipid-lowering drugs.

^*^ p < 0.05 compared with the group of lean without NAFLD;

^†^ p < 0.05 compared with the group of non-lean without NAFLD.

Supplementary Table 8. Associations of NAFLD with cardio-metabolic risk profiles according to weight (23 kg/m^2^) status in women and men

|  | | Crude OR | | | | Adjusted OR ^a^ | | |
| --- | --- | --- | --- | --- | --- | --- | --- | --- |
|  | Non-lean non-NAFLD | | Non-lean NAFLD | Lean NAFLD | Non-lean non-NAFLD | | Non-lean NAFLD | Lean NAFLD |
| **Women** |  | |  |  |  | |  |  |
| BP ≥ 130/80 mmHg | 1.34 (0.95, 1.89) | | 2.03 (1.45, 2.85) | 1.07 (0.67, 1.72) | 0.95 (0.62, 1.45) | | 1.33 (0.85, 2.10) | 0.97 (0.57, 1.66) |
| HbA1c ≥ 7.0% | 0.67 (0.45, 1.00) | | 2.06 (1.32, 3.23) | 2.47 (1.17, 5.22) | 0.62 (0.38, 1.02) | | 2.07 (1.16, 3.70) | 2.24 (0.99, 5.03) |
| TG ≥ 150mg/dL | 1.40 (1.00, 1.95) | | 3.18 (2.28, 4.43) | 3.54 (2.11, 5.92) | 1.17 (0.78, 1.74) | | 2.45 (1.59, 3.76) | 3.57 (2.02, 6.30) |
| LDL-C ≥ 100mg/dL | 0.78 (0.56, 1.09) | | 1.23 (0.89, 1.71) | 1.40 (0.86, 2.29) | 0.91 (0.61, 1.37) | | 1.49 (0.96, 2.30) | 1.59 (0.92, 2.77) |
| HDL-C ≤ 40/50 mg/dL for men/women | 1.54 (1.08, 2.19) | | 3.42 (2.35, 5.00) | 2.34 (1.35, 4.06) | 1.21 (0.78, 1.87) | | 1.89 (1.16, 3.06) | 1.78 (0.99,3.20) |
| HFpEF | 1.07 (0.66, 1.74) | | 1.23 (0.79, 1.92) | 2.10 (1.17, 3.75) | 1.00 (0.56, 1.77) | | 1.24 (0.69, 2.23) | 1.99 (1.04, 3.79) |
| LVH | 1.80 (1.21, 2.68) | | 1.22 (0.83, 1.81) | 1.47 (0.84, 2.56) | 1.30 (0.80, 2.13) | | 0.96 (0.57, 1.62) | 1.46 (0.77, 2.77) |
| **Men** |  | |  |  |  | |  |  |
| BP ≥ 130/80 mmHg | 1.78 (1.32, 2.39) | | 2.57 (1.94, 3.41) | 1.54 (0.95, 2.49) | 1.22 (0.86, 1.75) | | 1.73 (1.19, 2.50) | 1.33 (0.79, 2.24) |
| HbA1c ≥ 7.0% | 1.19 (0.84, 1.68) | | 2.21 (1.56, 3.13) | 1.75 (0.93, 3.27) | 1.07 (0.69, 1.65) | | 1.72 (1.08, 2.75) | 1.33 (0.68, 2.59) |
| TG ≥ 150mg/dL | 1.69 (1.26, 2.26) | | 5.19 (3.9, 6.93) | 4.41 (2.61, 7.45) | 1.73 (1.26, 2.37) | | 4.33 (3.16, 5.94) | 4.15 (2.38, 7.26) |
| LDL-C ≥ 100mg/dL | 1.35 (1.01, 1.81) | | 1.61 (1.23, 2.10) | 1.24 (0.78, 1.96) | 1.60 (1.16, 2.20) | | 1.51 (1.11, 2.06) | 0.96 (0.58, 1.59) |
| HDL-C ≤ 40/50 mg/dL for men/women | 1.41 (1.06, 1.88) | | 2.98 (2.25, 3.94) | 2.42 (1.46, 3.99) | 1.48 (1.08, 2.03) | | 2.63 (1.93, 3.58) | 2.20 (1.29, 3.73) |
| HFpEF | 1.11 (0.76, 1.63) | | 1.36 (0.96, 1.93) | 1.19 (0.65, 2.16) | 0.96 (0.63, 1.47) | | 1.43 (0.95, 2.14) | 1.34 (0.71, 2.54) |
| LVH | 2.09 (1.19, 3.67) | | 0.99 (0.56, 1.76) | 0.55 (0.16, 1.91) | 1.39 (0.75, 2.55) | | 0.91 (0.48, 1.71) | 0.73 (0.20, 2,62) |

BP, blood pressure; HbA1c, glycated hemoglobin; TG, triglycerides; LDL-C, low density lipoprotein cholesterol; HDL-C, high density lipoprotein cholesterol; HFpEF, heart failure with preserved ejection fraction; LVH, left ventricular hypertrophy.

Data are presented as odds ratios (95% confidence interval)

^a^ Models were adjusted for age, waist circumference, smoking status, HbA1c, BP, LDL-C, duration of T2DM, anti-diabetic drugs, anti-hypertensive drugs, and lipid-lowering drugs.

Supplementary Table 9. Adjusted means of cardio-metabolic risk factors according to obesity and NAFLD status after excluding patients taking TZDs, GLP-1RAs, and/or SGLT-2Is^a^

|  | Without NAFLD | | NAFLD | |
| --- | --- | --- | --- | --- |
|  | Non-obese | Obese | Obese | Non-obese |
| **Women** | n = 373 | n = 123 | n = 217 | n = `184 |
| Systolic BP (mmHg) | 134 (128, 139) | 137 (131, 143) | 134 (129, 140) | 133 (128, 138) |
| Diastolic BP (mmHg) | 77.6 (74.3, 80.8) | 78.3 (74.7, 81.9) | 81.1 (77.6, 84.5) ^*^ | 79.1 (75.9, 82.4) |
| FPG (mmol/L) | 8.58 (7.44, 9.71) | 7.96 (6.68, 9.23) | 8.84 (7.60, 10.07) | 8.95 (7.85, 10.06) |
| HbA1c (%) | 8.77 (8.14, 9.40) | 8.25 (7.54, 8.96) | 9.30 (8.63, 9.98) ^†^ | 9.54 (8.91, 10.17) ^*†^ |
| TC (mg/dL) | 158 (145, 170) | 147 (133, 160) | 162 (149, 175) ^†^ | 170 (157, 182) ^*†^ |
| TG (mg/dL) | 187 (140, 233) | 168 (116, 220) | 229 (179, 279) ^†^ | 275 (228, 321) ^*†^ |
| LDL-C (mg/dL) | 94.2 (84.1, 104.2) | 86.3 (75.1, 97.5) | 96.8 (86.0, 107.6) | 97.4 (87.4, 107.5) |
| HDL-C (mg/dL) | 44.4 (41.5, 47.4) | 41.1 (37.7, 44.4) ^*^ | 40.5 (37.3, 43.7) ^*^ | 40.5 (37.6, 43.5) ^*^ |
| Non-HDL-C (mg/dL) | 114 (102.8, 126) | 105 (92.3, 118) | 121 (109.0, 134) ^†^ | 129 (117.3, 140) ^*†^ |
| **Men** | n = 468 | n = 245 | n = 530 | n = 262 |
| Systolic BP (mmHg) | 130 (128, 132) | 132 (129, 135) | 132 (130, 134) | 129 (126, 132) |
| Diastolic BP (mmHg) | 80.8 (79.3, 82.4) | 82.6 (80.8, 84.3) | 84.1 (82.7, 85.5) ^*^ | 83.1 (81.4, 84.8) |
| FPG (mmol/L) | 8.45 (7.84, 9.05) | 9.03 (8.30, 9.76) | 9.49 (8.93, 10.06) | 8.61 (7.92, 9.29) |
| HbA1c (%) | 9.61 (9.31, 9.90) | 9.41 (9.07, 9.74) | 9.49 (9.23, 9.76) | 9.58 (9.25, 9.92) |
| TC (mg/dL) | 158 (152, 164) | 163 (157, 170) | 176 (170, 181) ^*†^ | 170 (164, 177) ^*^ |
| TG (mg/dL) | 211 (177, 244) | 253 (215, 290) | 354 (324, 384) ^*†^ | 322 (284, 360) ^*†^ |
| LDL-C (mg/dL) | 96.1 (91.5, 101) | 96.9 (91.8, 102) | 97.4 (93.3, 101) | 99.8 (94.6, 105) |
| HDL-C (mg/dL) | 38.6 (37.4, 39.8) | 37.6 (36.3, 38.9) | 35.4 (34.3, 36.4) ^*†^ | 35.1 (33.8, 36.4) ^*†^ |
| Non-HDL-C (mg/dL) | 119 (113, 125) | 126 (119, 132) | 140 (135, 146) ^*†^ | 134 (128, 141) ^*^ |

BP, blood pressure; FPG: fasting plasma glucose; HbA1c, glycated hemoglobin; TC, total cholesterol; TG, triglycerides; LDL-C, low density lipoprotein cholesterol; HDL-C, high density lipoprotein cholesterol.

Data are presented as means (95% confidence intervals).

^a^ Data were adjusted for age, waist circumference, smoking status, anti-diabetic drugs, anti-hypertensive drugs, and lipid-lowering drugs.

^*^ p < 0.05 compared with the group of non-obese without NAFLD;

^†^ p < 0.05 compared with the group of obesity without NAFLD.

Supplementary Table 10. Associations of NAFLD with cardio-metabolic risk profiles according to obesity status after excluding patients taking TZDs, GLP-1RAs, and/or SGLT-2Is^a^

|  | | Crude OR | | | | Adjusted OR ^a^ | | |
| --- | --- | --- | --- | --- | --- | --- | --- | --- |
|  | Obese non-NAFLD | | Obese NAFLD | Non-obese NAFLD | Obese non-NAFLD | | Obese NAFLD | Non-obese NAFLD |
| **Women** |  | |  |  |  | |  |  |
| BP ≥ 130/80 mmHg | 1.41 (0.91,2.17) | | 2.16 (1.47,3.15) | 1.12 (0.78,1.62) | 0.98 (0.58, 1.67) | | 1.34 (0.80, 2.24) | 1.02 (0.67, 1.56) |
| HbA1c ≥ 7.0% | 0.71 (0.45,1.12) | | 2.7 (1.61,4.5) | 2.53 (1.48,4.31) | 0.64 (0.36, 1.14) | | 2.97 (1.49, 5.91) | 2.46 (1.38, 4.39) |
| TG ≥ 150mg/dL | 1.48 (0.98,2.24) | | 3.09 (2.15,4.45) | 3.09 (2.1,4.53) | 1.19 (0.73, 1.95) | | 2.26 (1.39, 3.67) | 2.95 (1.93, 4.53) |
| LDL-C ≥ 100mg/dL | 0.63 (0.41,0.95) | | 1.31 (0.92,1.86) | 1.09 (0.76,1.57) | 0.71 (0.43, 1.16) | | 1.32 (0.81, 2.14) | 1.22 (0.80, 1.85) |
| HDL-C ≤ 40/50 mg/dL for men/women | 2.67 (1.61,4.44) | | 4.24 (2.67,6.73) | 2.39 (1.57,3.64) | 1.67 (0.93, 3.01) | | 1.99 (1.11, 3.57) | 1.67 (1.05, 2.64) |
| HFpEF | 1.45 (0.83,2.53) | | 1.16 (0.72,1.87) | 1.94 (1.22,3.07) | 1.90 (0.99, 3.65) | | 1.49 (0.78, 2.84) | 2.13 (1.25, 3.60) |
| LVH | 1.37 (0.86,2.17) | | 1.07 (0.73,1.58) | 0.82 (0.53,1.27) | 1.16 (0.66, 2.03) | | 0.96 (0.55, 1.67) | 0.80 (0.48, 2.12) |
| **Men** |  | |  |  |  | |  |  |
| BP ≥ 130/80 mmHg | 1.68 (1.2,2.35) | | 2.38 (1.8,3.15) | 1.38 (1,1.91) | 1.13 (0.76, 1.69) | | 1.66 (1.13, 2.42) | 1.21 (0.85, 1.72) |
| HbA1c ≥ 7.0% | 1.1 (0.74,1.62) | | 2.41 (1.67,3.47) | 1.84 (1.19,2.83) | 1.05 (0.65, 1.70) | | 1.88 (1.14, 3.10) | 1.41 (0.88, 2.27) |
| TG ≥ 150 mg/dL | 1.69 (1.24,2.32) | | 4.98 (3.75,6.6) | 3.31 (2.38,4.61) | 1.51 (1.03, 2.21) | | 3.32 (2.29, 4.82) | 2.79 (1.95, 3.99) |
| LDL-C ≥ 100 mg/dL | 1.11 (0.81,1.52) | | 1.21 (0.94,1.56) | 1.47 (1.08,2) | 1.29 (0.89, 1.88) | | 1.10 (0.78, 1.55) | 1.19 (0.85, 1.68) |
| HDL-C ≤ 40/50 mg/dL for men/women | 1.03 (0.62,1.71) | | 2.43 (1.56,3.79) | 1.41 (0.86,2.31) | 1.05 (0.72, 1.54) | | 1.96 (1.36, 2.83) | 1.46 (1.03, 2.08) |
| HFpEF | 0.96 (0.63,1.46) | | 1.19 (0.86,1.64) | 1.47 (1.01,2.15) | 0.87 (0.53, 1.43) | | 1.34 (0.87, 2.09) | 1.61 (1.06, 2.44) |
| LVH | 1.98 (1.18,3.31) | | 0.91 (0.55,1.5) | 0.36 (0.16,0.83) | 1.26 (0.66, 2.39) | | 0.89 (0.45, 1.73) | 0.55 (0.23, 1.31) |

BP, blood pressure; HbA1c, glycated hemoglobin; TG, triglycerides; LDL-C, low density lipoprotein cholesterol; HDL-C, high density lipoprotein cholesterol; HFpEF, heart failure with preserved ejection fraction; LVH, left ventricular hypertrophy.

Data are presented as odds ratios (95% confidence intervals).

^a^ Models were adjusted for age, waist circumference, smoking status, HbA1c, BP, LDL-C, duration of T2DM, anti-diabetic drugs, anti-hypertensive drugs, and lipid-lowering drugs.

Supplementary Table 11. Adjusted means of cardio-metabolic risk factors according to obesity and NAFLD status in women and men with T2DM duration ≥ 5 years^a^

|  | Without NAFLD | | NAFLD | |
| --- | --- | --- | --- | --- |
|  | Non-obese | Obese | Obese | Non-obese |
| **Women** | n = 254 | n = 96 | n = 113 | n = 110 |
| Systolic BP (mmHg) | 134 (128, 140) | 140 (133, 146) | 139 (133, 146) | 135 (129, 141) |
| Diastolic BP (mmHg) | 75.7 (72.3, 79.0) | 77.5 (73.7, 81.3) | 79.9 (76.1, 83.8) | 77.7 (74.3, 81.1) |
| FPG (mmol/L) | 9.20 (7.99, 10.41) | 8.13 (6.81, 9.44) | 8.95 (7.58, 10.33) | 9.20 (8.07, 10.32) |
| HbA1c (%) | 9.04 (8.45, 9.63) | 8.63 (7.96, 9.30) | 9.03 (8.34, 9.72) | 9.58 (8.98, 10.18) ^†^ |
| TC (mg/dL) | 160 (147, 173) | 151 (137, 166) | 157 (142, 172) | 165 (152, 178) |
| TG (mg/dL) | 217 (166, 268) | 182 (124, 239) | 249 (190, 308) ^†^ | 293 (241, 344) ^*†^ |
| LDL-C (mg/dL) | 91.6 (81.1, 102.0) | 84.5 (72.7, 96.3) | 89.3 (77.2, 101.3) | 90.2 (79.7, 100.7) |
| HDL-C (mg/dL) | 45.7 (42.3, 49.0) | 44.7 (41.0, 48.5) ^*^ | 42.1 (38.3, 46.0) | 39.1 (35.7, 42.5) ^*†^ |
| Non-HDL-C (mg/dL) | 115 (102.0, 128) | 106 (91.4, 120) | 115 (100.5, 130) | 126 (113.3, 139) ^†^ |
| **Men** | n = 294 | n = 180 | n = 222 | n = `102 |
| Systolic BP (mmHg) | 130 (126, 133) | 132 (129, 136) | 131 (128, 135) | 128 (124, 132) |
| Diastolic BP (mmHg) | 78.2 (76.3, 80) | 81.0 (79.0, 83) | 82.0 (80.1, 84) ^*^ | 82.5 (79.9, 85) ^*^ |
| FPG (mmol/L) | 8.06 (7.48, 8.64) | 8.38 (7.66, 9.10) | 8.93 (8.28, 9.58) | 8.66 (7.84, 9.49) |
| HbA1c (%) | 8.96 (8.64, 9.28) | 8.52 (8.17, 8.87) | 8.72 (8.39, 9.06) | 8.87 (8.42, 9.32) |
| TC (mg/dL) | 153 (146, 160) | 155 (148, 163) | 164 (156, 171) | 172 (161, 182) ^*†^ |
| TG (mg/dL) | 197 (162, 232) | 211 (173, 249) | 306 (269, 342) ^*†^ | 331 (282, 380) ^*†^ |
| LDL-C (mg/dL) | 92.3 (86.6, 98.0) | 93.7 (87.5, 99.9) | 91.9 (85.9, 97.8) | 98.8 (90.8, 106.9) |
| HDL-C (mg/dL) | 39.9 (38.3, 41.5) | 39.1 (37.4, 40.9) | 37.2 (35.6, 38.9) | 35.1 (33.8, 36.4) ^*^ |
| Non-HDL-C (mg/dL) | 113 (106, 120) | 117 (109, 124) | 126 (119, 134) ^*^ | 134 (124, 144) ^*†^ |

BP, blood pressure; FPG: fasting plasma glucose; HbA1c, glycated hemoglobin; TC, total cholesterol; TG, triglycerides; LDL-C, low density lipoprotein cholesterol; HDL-C, high density lipoprotein cholesterol.

Data are presented as means (95% confidence intervals).

^a^ Data were adjusted for age, waist circumference, smoking status, anti-diabetic drugs, anti-hypertensive drugs, and lipid-lowering drugs.

^*^ p < 0.05 compared with the group of non-obese without NAFLD;

^†^ p < 0.05 compared with the group of obesity without NAFLD.

Supplementary Table 12. Associations of NAFLD with cardio-metabolic risk profiles according to obesity status in women and men with T2DM duration ≥ 5 years^a^

|  | | Crude OR | | | | Adjusted OR ^a^ | | |
| --- | --- | --- | --- | --- | --- | --- | --- | --- |
|  | Obese non-NAFLD | | Obese NAFLD | Non-obese NAFLD | Obese non-NAFLD | | Obese NAFLD | Non-obese NAFLD |
| **Women** |  | |  |  |  | |  |  |
| BP ≥ 130/80 mmHg | 1.21 (0.74,1.99) | | 1.46 (0.9,2.37) | 1.29 (0.8,2.08) | 1.04 (0.57, 1.92) | | 1.24 (0.65, 2.37) | 1.32 (0.76, 2.32) |
| HbA1c ≥ 7.0% | 0.83 (0.47,1.46) | | 2.86 (1.35,6.05) | 3.64 (1.59,8.33) | 0.54 (0.26, 1.11) | | 1.90 (0.76, 4.75) | 2.73 (1.16, 6.44) |
| TG ≥ 150mg/dL | 1.18 (0.74,1.9) | | 3.86 (2.32,6.43) | 3.93 (2.34,6.59) | 1.04 (0.58,1.86) | | 3.07 (1.60, 5.88) | 3.84 (2.15, 6.87) |
| LDL-C ≥ 100mg/dL | 0.54 (0.34,0.88) | | 0.93 (0.6,1.47) | 0.83 (0.53,1.3) | 0.56 (0.31, 1.00) | | 0.99 (0.54, 1.82) | 0.97 (0.58, 1.64) |
| HDL-C ≤ 40/50 mg/dL for men/women | 1.17 (0.61,2.23) | | 4.34 (2.42,7.81) | 3.22 (1.87,5.56) | 1.04 (0.55, 1.96) | | 1.95 (0.96, 3.94) | 2.28 (1.24, 4.17) |
| HFpEF | 1.45 (0.83,2.53) | | 0.78 (0.4,1.51) | 1.45 (0.8,2.61) | 1.17 (0.54, 2.54) | | 0.72 (0.30, 1.73) | 1.50 (0.76, 2.93) |
| LVH | 1.42 (0.84,2.4) | | 1.27 (0.78,2.09) | 0.91 (0.54,1.56) | 1.40 (0.73, 2.67) | | 1.22 (0.62, 2.41) | 0.89 (0.47, 1.66) |
| **Men** |  | |  |  |  | |  |  |
| BP ≥ 130/80 mmHg | 1.84 (1.23,2.77) | | 2.36 (1.59,3.51) | 1.49 (0.92,2.42) | 1.33 (0.81, 2.19) | | 1.78 (1.07, 2.98) | 1.27 (0.75, 2.15) |
| HbA1c ≥ 7.0% | 1.02 (0.65,1.61) | | 1.43 (0.91,2.25) | 1.74 (0.93,3.27) | 0.96 (0.54, 1.69) | | 1.17 (0.64, 2.13) | 1.49 (0.76, 2.90) |
| TG ≥ 150 mg/dL | 1.76 (1.21,2.58) | | 4.82 (3.24,7.15) | 3.66 (2.23,6.02) | 1.56 (0.96, 2.53) | | 3.30 (1.99, 5.46) | 3.33 (1.93, 5.72) |
| LDL-C ≥ 100 mg/dL | 0.98 (0.67,1.44) | | 1.02 (0.72,1.45) | 1.73 (1.09,2.73) | 1.00 (0.62, 1.61) | | 0.91 (0.57, 1.46) | 1.50 (0.91, 2.48) |
| HDL-C ≤ 40/50 mg/dL for men/women | 1.18 (0.81,1.72) | | 1.95 (1.35,2.81) | 1.35 (0.85,2.15) | 0.99 (0.63, 1.58) | | 1.52 (0.94, 2.43) | 1.16 (0.71, 1.91) |
| HFpEF | 1.04 (0.65,1.67) | | 1.43 (0.94,2.17) | 0.96 (0.54,1.72) | 1.13 (0.63, 2.04) | | 1.63 (0.93, 2.86) | 1.02 (0.55, 1.91) |
| LVH | 2.27 (1.3,3.96) | | 1.07 (0.59,1.96) | 0.21 (0.05,0.89) | 1.32 (0.64, 2.71) | | 0.69 (0.32, 1.49) | 0.26 (0.06, 1.17) |

BP, blood pressure; HbA1c, glycated hemoglobin; TG, triglycerides; LDL-C, low density lipoprotein cholesterol; HDL-C, high density lipoprotein cholesterol; HFpEF, heart failure with preserved ejection fraction; LVH, left ventricular hypertrophy.

Data are presented as odds ratios (95% confidence intervals).

^a^ Models were adjusted for age, waist circumference, smoking status, HbA1c, BP, LDL-C, anti-diabetic drugs, anti-hypertensive drugs, and lipid-lowering drugs.

Supplementary Table 13. Adjusted means of cardio-metabolic risk factors according to obesity and NAFLD status in women and men with T2DM duration < 5 years^a^

|  | Without NAFLD | | NAFLD | |
| --- | --- | --- | --- | --- |
|  | Non-obese | Obese | Obese | Non-obese |
| **Women** | n = 157 | n = 50 | n = 133 | n = 92 |
| Systolic BP (mmHg) | 124 (115, 133) | 124 (114, 133) | 123 (114, 132) | 124 (115, 133) |
| Diastolic BP (mmHg) | 77.7 (71.9, 83.5) | 77.7 (71.4, 84.1) | 80.2 (74.3, 86.0) | 78.8 (72.8, 84.7) |
| FPG (mmol/L) | 9.04 (7.23, 10.9) | 8.67 (6.67, 10.7) | 10.03 (8.16, 11.9) | 10.01 (8.15, 11.9) |
| HbA1c (%) | 8.44 (7.16, 9.73) | 7.80 (6.40, 9.21) | 9.07 (7.78, 10.36) ^†^ | 9.33 (8.01, 10.64) ^†^ |
| TC (mg/dL) | 163 (140, 186) | 151 (126, 177) | 177 (153, 200) ^†^ | 185 (161, 209) ^*†^ |
| TG (mg/dL) | 236 (148, 325) | 231 (134, 329) | 279 (190, 369) | 337 (246, 428) ^*†^ |
| LDL-C (mg/dL) | 99.1 (80.9, 117) | 89.7 (69.7, 110) | 108.0 (89.6, 126) ^†^ | 109.1 (90.3, 128) ^†^ |
| HDL-C (mg/dL) | 45.7(40.4, 51.0) | 41.2 (35.4, 47.0) | 41.8 (36.5, 47.2) | 44.4 (38.9, 49.8) |
| Non-HDL-C (mg/dL) | 118 (97.4, 140) | 110 (86.6, 133) | 135 (114, 157) ^†^ | 141 (119, 162) ^*†^ |
| **Men** | n = 229 | n = 111 | n = 381 | n = `179 |
| Systolic BP (mmHg) | 131 (128, 135) | 130 (126, 133) | 132 (129, 134) | 131 (128, 134) |
| Diastolic BP (mmHg) | 85.3 (83.0, 87.7) | 84.3 (81.7, 86.8) | 86.4 (84.5, 88.2) | 85.3 (83.0, 87.6) |
| FPG (mmol/L) | 8.28 (7.23, 9.34) | 8.88 (7.71, 10.04) | 9.47 (8.61, 10.34) | 8.62 (7.56, 9.68) |
| HbA1c (%) | 9.62 (9.14, 10.1) | 9.62 (9.10, 10.1) | 9.72 (9.33, 10.1) | 9.88 (9.40, 10.4) |
| TC (mg/dL) | 165 (156, 174) | 167 (157, 178) | 184 (176, 191) ^*†^ | 175 (166, 184) |
| TG (mg/dL) | 243 (186, 301) | 299 (236, 362) | 395 (349, 442) ^*†^ | 345 (288, 402) ^*^ |
| LDL-C (mg/dL) | 100.1 (93.1, 107) | 99.7 (92.1, 107) | 101.4 (95.8, 107) | 103.2 (96.2, 110) |
| HDL-C (mg/dL) | 38.0 (36.4, 39.6) | 36.4 (34.6, 38.1) | 33.9 (32.6, 35.2) ^*†^ | 34.1 (32.5, 35.6) ^*^ |
| Non-HDL-C (mg/dL) | 127 (117, 136) | 131 (121, 141) | 149 (142, 157) ^*†^ | 140 (131, 149) ^*^ |

BP, blood pressure; FPG: fasting plasma glucose; HbA1c, glycated hemoglobin; TC, total cholesterol; TG, triglycerides; LDL-C, low density lipoprotein cholesterol; HDL-C, high density lipoprotein cholesterol.

Data are presented as means (95% confidence intervals).

^a^ Data were adjusted for age, waist circumference, smoking status, anti-diabetic drugs, anti-hypertensive drugs, and lipid-lowering drugs.

^*^ p < 0.05 compared with the group of non-obese without NAFLD;

^†^ p < 0.05 compared with the group of obesity without NAFLD.

Supplementary Table 14. Associations of NAFLD with cardio-metabolic risk profiles according to obesity status in women and men with T2DM duration < 5 years^a^

|  | | Crude OR | | | | Adjusted OR ^a^ | | |
| --- | --- | --- | --- | --- | --- | --- | --- | --- |
|  | Obese non-NAFLD | | Obese NAFLD | Non-obese NAFLD | Obese non-NAFLD | | Obese NAFLD | Non-obese NAFLD |
| **Women** |  | |  |  |  | |  |  |
| BP ≥ 130/80 mmHg | 1.88 (0.95,3.71) | | 3.65 (2.12,6.29) | 1.2 (0.71,2.02) | 1.29 (0.55, 3.07) | | 1.60 (0.75, 3.43) | 1.00 (0.54, 1.84) |
| HbA1c ≥ 7.0% | 0.44 (0.23,0.87) | | 2 (1.08,3.72) | 1.93 (0.96,3.87) | 0.62 (0.27, 1.43) | | 2.59 (1.07, 6.28) | 1.93 (0.89, 4.17) |
| TG ≥ 150mg/dL | 1.76 (0.92,3.35) | | 2.61 (1.61,4.23) | 2.2 (1.29,3.75) | 1.23 (0.59, 2.59) | | 1.55 (0.81, 2.97) | 2.02 (1.13, 3.62) |
| LDL-C ≥ 100mg/dL | 0.8 (0.42,1.53) | | 2.08 (1.26,3.42) | 1.8 (1.04,3.13) | 1.00 (0.46, 2.18) | | 2.33 (1.14, 4.75) | 1.99 (1.06, 3.76) |
| HDL-C ≤ 40/50 mg/dL for men/women | 2.67 (1.12,6.36) | | 3.14 (1.68,5.86) | 1.37 (0.76,2.48) | 1.94 (0.74, 5.09) | | 2.00 (0.87, 4.60) | 1.01 (0.53, 1.91) |
| HFpEF | 1.59 (0.63,3.96) | | 1.83 (0.94,3.57) | 2.71 (1.35,5.42) | 1.90 (0.99, 3.65) | | 1.49 (0.78, 2.84) | 2.13 (1.25, 3.60) |
| LVH | 1.41 (0.65,3.04) | | 1.13 (0.63,2.02) | 0.97 (0.5,1.89) | 1.00 (0.41, 2.47) | | 0.82 (0.36, 1.88) | 0.98 (0.47, 2.07) |
| **Men** |  | |  |  |  | |  |  |
| BP ≥ 130/80 mmHg | 1.2 (0.75,1.94) | | 2.16 (1.5,3.1) | 1.47 (0.97,2.23) | 0.70 (0.40, 1.24) | | 1.28 (0.77, 2.10) | 1.24 (0.78, 1.97) |
| HbA1c ≥ 7.0% | 1.34 (0.75,2.4) | | 2.65 (1.67,4.22) | 2.19 (1.25,3.84) | 1.34 (0.66, 2.72) | | 2.53 (1.31, 4.88) | 1.66 (0.90, 3.04) |
| TG ≥ 150 mg/dL | 1.83 (1.15,2.91) | | 5.11 (3.52,7.4) | 3.1 (2.03,4.74) | 1.44 (0.83, 2.48) | | 3.45 (2.11, 5.65) | 2.48 (1.58, 3.90) |
| LDL-C ≥ 100 mg/dL | 1.39 (0.87,2.21) | | 1.18 (0.85,1.65) | 1.27 (0.85,1.9) | 1.59 (0.93, 2.71) | | 1.28 (0.82, 2.00) | 1.15 (0.74, 1.77) |
| HDL-C ≤ 40/50 mg/dL for men/women | 1.72 (1.07,2.76) | | 4.46 (3.05,6.52) | 2.24 (1.47,3.42) | 1.37 (0.79, 2.40) | | 2.93 (1.77, 4.86) | 1.90 (1.20, 2.99) |
| HFpEF | 1.03 (0.55,1.95) | | 1.23 (0.78,1.94) | 1.78 (1.07,2.94) | 2.09 (0.73, 6.00) | | 2.52 (1.01, 6.28) | 2.68 (1.23, 5.87) |
| LVH | 0.66 (0.21,2.1) | | 0.91 (0.43,1.92) | 0.62 (0.23,1.68) | 0.56 (0.15, 2.10) | | 1.13 (0.39, 3.25) | 0.77 (0.26, 2.29) |

BP, blood pressure; HbA1c, glycated hemoglobin; TG, triglycerides; LDL-C, low density lipoprotein cholesterol; HDL-C, high density lipoprotein cholesterol; HFpEF, heart failure with preserved ejection fraction; LVH, left ventricular hypertrophy.

Data are presented as odds ratios (95% confidence intervals).

^a^ Models were adjusted for age, waist circumference, smoking status, HbA1c, BP, LDL-C, anti-diabetic drugs, anti-hypertensive drugs, and lipid-lowering drugs.
